# P23 Acts as Functional RBP in the Macrophage Inflammation Response

**DOI:** 10.3389/fmolb.2021.625608

**Published:** 2021-06-11

**Authors:** Sebastian de Vries, Vladimir Benes, Isabel S. Naarmann-de Vries, Cornelia Rücklé, Katharina Zarnack, Gernot Marx, Dirk H. Ostareck, Antje Ostareck-Lederer

**Affiliations:** ^1^ Department of Intensive Care Medicine, University Hospital RWTH Aachen, Aachen, Germany; ^2^ Genomics Core Facility, European Molecular Biology Laboratory (EMBL), Heidelberg, Germany; ^3^ Buchmann Institute of Molecular Life Science, Goethe University Frankfurt, Frankfurt, Germany

**Keywords:** macrophage inflammatory response, RNA-binding protein, P23/PTGES3, *Kif15* mRNA, migration, phagocytosis, post-transcriptional control of gene expression

## Abstract

Macrophages exert the primary cellular immune response. Pathogen components like bacterial lipopolysaccharides (LPS) stimulate macrophage migration, phagocytotic activity and cytokine expression. Previously, we identified the poly(A)^+^ RNA interactome of RAW 264.7 macrophages. Of the 402 RNA-binding proteins (RBPs), 32 were classified as unique in macrophages, including nineteen not reported to interact with nucleic acids before. Remarkably, P23 a HSP90 co-chaperone, also known as cytosolic prostaglandin E2 synthase (PTGES3), exhibited differential poly(A)^+^ RNA binding in untreated and LPS-induced macrophages. To identify mRNAs bound by P23 and to elucidate potential regulatory RBP functions in macrophages, we immunoprecipitated P23 from cytoplasmic extracts of cross-linked untreated and LPS-induced cells. RNAseq revealed that enrichment of 44 mRNAs was reduced in response to LPS. *Kif15* mRNA, which encodes kinesin family member 15 (KIF15), a motor protein implicated in cytoskeletal reorganization and cell mobility was selected for further analysis. Noteworthy, phagocytic activity of LPS-induced macrophages was enhanced by P23 depletion. Specifically, in untreated RAW 264.7 macrophages, decreased P23 results in *Kif15* mRNA destabilization, diminished KIF15 expression and accelerated macrophage migration. We show that the unexpected RBP function of P23 contributes to the regulation of macrophage phagocytotic activity and migration.

## Introduction

As primary responding cells of the innate immune system, macrophages recognize pathogen components, like bacterial lipopolysaccharides (LPS) by Toll-like receptor 4 (TLR4). Different branches of TLR4 signaling pathways involve mitogen activated protein kinases (MAPK) and nuclear factor kappa B (NFκB) and ultimately induce inflammatory cytokine and chemokine expression, macrophage migration and phagocytotic activity (Zanotti and Kumar, 2002; Medzhitov and Horng, 2009; Smale, 2012; Vaure and Liu, 2014). Cytokines and chemokines are essential to coordinate cellular responses to infection, but their excessive synthesis disturbs the delicate balance between pro- and anti-inflammatory processes, leading to systemic capillary leakage, tissue destruction, and lethal organ failure (Zanotti and Kumar, 2002; Hotchkiss and Karl, 2003). LPS-induced changes in gene expression (Rutledge et al., 2011; Reynier et al., 2012) require downstream post-transcriptional checkpoints to safeguard faultless immune responses (Carpenter et al., 2014; Kafasla et al., 2014). Emerging evidence highlights the role of RNA-binding proteins (RBPs) in the regulation of both, mRNA turnover and mRNA translation to execute coordinated protein synthesis required for rapid and purposive macrophage functions (Ostareck and Ostareck-Lederer, 2019). Specific RBPs, namely Tristetraprolin (TTP), human antigen R (HUR), T-cell intracellular antigen 1 related protein (TIAR) and heterogeneous ribonucleoprotein K (HNRNPK) are known to modulate the activation and resolution of macrophage immune reactions [reviewed in Ostareck and Ostareck-Lederer (2019)]. Systematic analyses of LPS-controlled RBP-RNA interactions in macrophages revealed first insights into the complex regulatory RNA-protein interactome. Based on their domain composition RBPs exhibit affinities for specific AU-rich or U-rich motifs like TTP (Stoecklin et al., 2008; Kratochvill et al., 2011; Sedlyarov et al., 2016; Tiedje et al., 2016), HUR (Sedlyarov et al., 2016) and TIAR (Kharraz et al., 2016) or pyrimidine-rich sequences, like HNRNPK (Liepelt et al., 2014). They target mRNAs that encode signaling checkpoint proteins, control their stability and timely translation, and confer the regulation of macrophage activity.

Employing an RNA interactome capture approach (Castello et al., 2013) we comprehensively identified RBPs, which modulate the LPS response of RAW 264.7 macrophages (Liepelt et al., 2016). The macrophage poly(A)^+^ RNA interactome consists of 402 RBPs, including 91 proteins previously not annotated as RBPs (Liepelt et al., 2016). In a comparison with other studies (Baltz et al., 2012; Castello et al., 2012; Kwon et al., 2013; Liao et al., 2016), 32 macrophage-specific RBPs were disclosed, including 19 proteins functionally unrelated to RNA. Interestingly, among them we identified P23, a HSP90 co-chaperone (Johnson et al., 1994), as a new RBP.

P23 facilitates the ATP-driven HSP90 binding to client proteins, for example progesterone receptor (Johnson et al., 1994), Fes tyrosine kinase and transcription factor Hsf1 (Nair et al., 1996), heme-regulated kinase HRI (Xu et al., 1997), polymerases such as telomerase (Holt et al., 1999) and viral reverse transcriptase (Hu et al., 2002), [reviewed in Felts and Toft (2003)]. A genomic and proteomic screen in yeast revealed a network that displayed known HSP90 client-independent functions of P23/Sba1 and demonstrated features in ribosome biogenesis and vesicle-mediated transport (Echtenkamp et al., 2011). Moreover, P23 is known as cytosolic prostaglandin E2 synthase 3 (PTGES3) converting COX-derived prostaglandin H2 to E2 (Tanioka et al., 2000). Eicosanoid signaling is critical in the control of peritoneal macrophage gene expression (Gautier et al., 2012) and PTGES3 activity is elevated in response to LPS (Naraba et al., 1998).

In this report, we uncovered an unexpected function of P23 as a post-transcriptional regulator in untreated macrophages. The differential binding of P23 to the mRNA encoding kinesin family member 15 (KIF15) and its impact on *Kif15* mRNA stability, suggests a function in the regulation of macrophage migration and phagocytosis. This novel mechanistic insight sheds light on the modulation of the inflammatory response and provides indications for molecular interventions.

## Materials and Methods

### Plasmids

PBSIIKS-LUC-pA-NB (Liepelt et al., 2014), pBSIISK-10R (Ostareck-Lederer et al., 1994) and pET 16b-P23 (Liepelt et al., 2016) have been described. *Kif15* mRNA 3′UTR (NM_010620.1) was PCR-amplified with Phusion High Fidelity DNA polymerase (Thermo Fischer Scientific, Carlsbad, CA, United States, F530S) from RAW 246.7 cell total RNA after reverse transcription employing Maxima H Minus First Strand cDNA Synthesis Kit (Thermo Fisher Scientific, K1682) and oligo-dT primers. Primers mur-*Kif15*-3utr-PstI (5′’-att​gta​agg​ctg​cag​GGA​TCC​CAG​CTA​TCT​TCA​TAC​AC-3′) and mur-*Kif15*-3utr-XhoI (5′tac​cgt​cga​cct​cga​gTG​TTT​TTA​AAA​AAG​ATT​TTA​TTT​GAA​AAA​CTG​GAC​ATGTAGAAAATGGC-3′) were used to generate a PCR product that was ligated into pBluescript II KS *via* XhoI/PstI.

### Cell Culture and Lipopolysaccharides Treatment

RAW 264.7 cells (ATCC, Wesel, Germany, TIB-71) were cultured in DMEM (Thermo Fisher Scientific, 12430054) supplemented with 10% heat inactivated FBS (Biochrom, Berlin, Germany, S0613), 1x penicillin/streptomycin (Thermo Fisher Scientific). For LPS treatment, *E.coli* LPS (serotype 0111:B4, Sigma-Aldrich, St. Louis, MO, United States) was added to the medium for 2, 6 and 24 h, as indicated.

### UV-Crosslinking

Cells on culture dishes were washed twice with ice-cold PBS, kept on ice and exposed to UV light (254 nm, 0.15 J/cm^2^) (Stratalinker 2400, Stratagene, La Jolla, CA) and harvested in ice-cold PBS (Liepelt et al., 2016).

### Lysate Preparation

Total cell lysate preparation was performed according to Ostareck-Lederer et al. (2002).

### Cytoplasmic Extract Preparation

Cytoplasmic extract was prepared as described in Liepelt et al. (2014).

### Western Blot Assays

Western blot assays were performed as described (Naarmann et al., 2010) with specific antibodies (Supplementary Material). Images were acquired with the LAS4000 system (GE Healthcare Chicago, IL, United States) and quantified using ImageQuant (GE Healthcare).

### P23 Immunoprecipitation

Immunoprecipitation from 1 mg cytoplasmic extract of crosslinked RAW 264.7 cells either untreated or 2 h LPS treated was performed with P23 antibodies or a non-related luciferase control antibody (Liepelt et al., 2016) in three independent biological replicates. Briefly, crosslinked cells were centrifuged (5 min, 500 × *g*, 4°C) snap frozen in liquid nitrogen and lyzed in 1 vol. IP buffer (20 mM HEPES pH 7.4, 100 mM KCl, 5 mM MgAc, 0.025% Triton X-100, 1 mM DTT, 1 mM PMSF and 1 μg/ml Leupeptin) by passing ten times through a 20G and subsequently 26G needle. Antibodies against P23: JJ3 (Figures 2, 7) (NB300-576, Novus Biological, Littleton, Colorado, United States) and JJ6 (Figure 7) (NB110-96879) as well as LUC (Figures 2, 7) (Luci17 sc-57604, Santa Cruz Biotechnology, Dallas, Texas, United States) were incubated overnight at 4°C with 40 µl Protein G-Sepharose (GE Healthcare Chicago, IL, United States, 17-0618-01). Antibody-coupled beads were incubated 2 h, 4°C with 1 mg cytoplasmic RAW 264.7 extract (IP buffer) from untreated or 10 ng/ml LPS stimulated cells. Beads were washed twice in IP buffer and applied to RNA isolation using Trizol (Thermo Fisher Scientific) (80%) and Western blotting (20%).

### RNA Preparation

For analysis of precipitated RNA covalently bound proteins were removed by proteinase K (Sigma-Aldrich, 311582). Samples from input and eluate were pre-incubated with 5×proteinase K buffer (50 mM Tris/HCl pH 7.5, 750 mM NaCl, 1% SDS, 50 mM EDTA, 2.5 mM DTT, 25 mM CaCl_2_) and 40U RiboLock (Thermo Fisher Scientific, EO0381) for 30 min at 65°C. After addition of 20 µg proteinase K, samples were incubated for 1 h at 50°C (Liepelt et al., 2016). For the analysis of *Kif15* mRNA stability, cells were treated with Actinomycin D (Sigma-Aldrich, A9415-2MG) (5 μg/ml) for 0, 1, 2, 3 and 4 h. RNA was extracted using Trizol (Thermo Fisher Scientific, 15596018). 100 pg *in vitro* transcribed cap-LUC-poly(A)^+^-mRNA was added as extraction control in Figures 2D, 4B, 5C. For reverse transcription, 1 µg total RNA, random primers and Maxima H Minus First Strand cDNA Synthesis Kit (Thermo Fisher Scientific, K1682) were used. Quantitative PCR (qPCR) was performed with PowerSYBRGreen (Thermo Fisher Scientific, 4368702) on a StepOnePlus PCR system (Thermo Fisher Scientific), primers are listed in the Supplementary Material. RNA levels were determined by the ΔΔC_t_ method (Livak and Schmittgen, 2001).

### RNA Sequencing

RNA samples from inputs or immunoprecipitations with P23 or Luciferase control antibodies (all samples from untreated RAW 264.7 cells or cells treated with LPS for 2 h) were analyzed with an RNA 6000 Nano Kit on a 2100 Bioanalyzer (Agilent Technologies; Santa Clara, CA, United States). Barcoded RNA-seq libraries were prepared from either pulled-down or high quality total RNA samples (25 ng/sample) using the Illumina TruSeq total RNA Sample Preparation v2 Kit. Obtained libraries that passed the QC step were combined in equimolar amounts into pools of seven libraries; 8pM solution of each pool was loaded per lane of the Illumina sequencer HiSeq 2000 flowcell and sequenced bi-directionally (each read 50 bases long, altogether 100 bases) with the Illumina v3 SBS chemistry. Sequencing generated >60 million of paired sequence reads/library (∼450 million of paired sequence reads/lane).

### Analysis of RNAseq Data

Trimming of low quality reads and adapters was performed using Trimmomatic v.0.33 (Bolger et al., 2014) with the following settings: ILLUMINACLIP:TruSeq3-PE-2.fa:2:30:10 LEADING:3 TRAILING:3 SLIDINGWINDOW:4:15 MINLEN:36. The data quality was evaluated using the FastQC software v0.11.2 before and after trimming. The mouse reference genome (version GRCm38/mm10) and gene annotation (Ensembl release 83) were downloaded from Ensembl. Mapping was performed using Tophat v.2.0.14 (Kim et al., 2013) with standard parameters and the additional annotation file. Quantification of unique read counts per gene was performed using HTSeq-0.6.1 (Anders et al., 2015) with default parameters except for stranded = no. Differential expression analysis was performed in R using edgeR (Robinson et al., 2010). Initial assessment of replicates by pairwise distance and principal component analysis (PCA) led to removal of one replicate (LUC 2 h replicate III) as apparent outlier. Comparison of the remaining data yielded 52 RNAs that were specifically enriched in the P23 IP compared to LUC IP at the 0 h time point (adjusted *p*-value ≤ 0.05, Benjamini Hochberg correction, log_2_-transformed fold change >0) (Benjamini and Hochberg, 1995). In order to assess changes in P23 binding to the 52 RNAs after LPS treatment, means of TPM values from the replicates were used to calculate ratios for P23/LUC at 0 and 2 h LPS treatment (Supplementary Table S1).

### RNA Motif Identification

Identified unbound RNA sequences were determined as follows: log2 FC < −0.2 and >0.1 in input 2 h over input 0 h were removed, as well as all transcripts with TPM = 0.00 in input 0 and/or 2 h. Furthermore, transcripts without KEGG-ID and/or MGI description were removed. Then, a minimal TPM was set to 10, and the transcripts were filtered for a log2 FC to < −0.1 in inputs 2 h/0 h. Finally, transcripts with log2 FC > 0.00 for P23-0 h vs. LUC-0 h immunoprecipitation were removed. 3′UTR sequences for P23-interacting mRNAs as well as unbound mRNAs were retrieved from the Ensembl genome database (version 92). Sequences were filtered for the presence of a RefSeq-ID. If more than one sequence was identified, the sequence with the best transcript support level was chosen. Discriminative motif enrichment analysis in the P23-interacting mRNAs was performed using MEME (Bailey et al., 2009). The 3′UTR sequences of unbound mRNAs were used as background input. The detected motif with the lowest *p*-value was resubmitted to MAST (Bailey and Gribskov, 1998) to rescreen all sequences for the presence of the motif. In order to assess the sequence composition of the *Kif15* 3′UTR, we moved a 30-nt sliding window along the *Kif15* 3′UTR (606nt, NM_010620.1) to count the frequency of U/G-only hexamers.

### *In vitro* Transcription

Linearized plasmids were transcribed using the T3 or T7 polymerase MEGAscript kit (Thermo Fisher Scientific, AM1338 or AM1334, respectively) following manufacturer’s protocols.

### Expression of Recombinant Proteins

His-P23 was expressed and prepared as described (Liepelt et al., 2016).

### *In vitro* RNA-Protein Binding Assay

*In vitro* transcribed *Kif15* mRNA 3′UTR (606nt) and ALOX15 mRNA 3′UTR differentiation control element (DICE) (250nt) were 3′biotinylated (Pierce RNA 3′end biotinylation kit, Thermo Scientific, #20160) following manufacturer’s protocols. Per reaction 12.5 µl streptavidin-magnetic beads (NEB, #S1420S) were washed three times with each 0.5 ml binding buffer (BB: 300 mM KCl, 20 mM Tris pH 8.0, 10% sucrose, 1 mM EDTA, 0.5% Triton X-100). Beads were incubated with 4 pmol biotinylated RNA and 20U Ribolock (Thermo Scientific, # EO 0382) in 0.5 ml BB, 30 min, at RT with rotation. Beads were isolated in a magnetic separation rack (NEB), washed twice in BB and subsequently incubated with 40 pmol His-P23, 20U Ribolock and 20 µl protease inhibitor (cOmplete, EDTA-fee protease inhibitor cocktail, Roche, # 04693132001) in 0.5 ml BB for 30 min at RT with rotation. Finally, beads were washed four times and denatured for Western blot analysis.

### RNA Interference

For RNAi, RAW 264.7 cells (1 × 10^6^ cells in DMEM without FBS and antibiotics) were transfected by electroporation at 0.36 kV and 500 µF (Gene Pulser-Xcell, Biorad, Hercules CA, United States) with 500 pmol siRNAs (MWG) (Supplementary Material), and cells were harvested 24 h post-transfection or further treated as indicated.

### Phagocytosis Assay

RAW 264.7 cells were transfected with P23-specific and control siRNAs for 4 h, prior to 24 h LPS treatment (100 ng/ml). Cells were incubated with Fluoresbrite® YG Carboxylate Microspheres, 0.50 μm (Polysciences, Warrington, PA, United States, 15700-10) for 1 h at 37°C and beads uptake was analyzed by immunofluorescence microscopy.

### Migration Assay

Cells transferred to Transwell chambers (Sigma-Aldrich, Costar, CLS3422) were subjected to starvation (1% FCS) for 24 h before they were exposed to LPS (100 ng/ml) at 10%FSC standard conditions. Following siRNA (Supplementary Material) transfection, cells were allowed to recover for 6 h under standard conditions prior to 16 h FCS starvation and LPS induction. Migrated cells were fixed on cover slips with 4% paraformaldehyde in PBS (Sigma-Aldrich, 33220), embedded in ProLong Gold Antifade Reagent with DAPI (Thermo Scientific, Carlsbad, CA, United States, P36931) and analyzed by fluorescence microscopy.

### Immunofluorescence and Fluorescence *in situ* Hybridization

Immunofluorescence staining was essentially performed as described in De Vries et al. (2013) with a specific P23 antibody (Supplementary Material) and FISH as in Staudacher et al. (2015) with a *Kif15* probe (Supplementary Material). Images were acquired with AxioVision on an Apotome 2 Microscope (Carl-Zeiss-Jena, Jena, Germany).

### Statistical Analysis

Experiments were recapitulated at least three times, statistical analysis was performed with one-way ANOVA, with significance levels defined as * = *p* < 0.05, ** = *p* < 0.01, *** = *p* < 0.001.

## Results

### Depletion of P23 Enhances Phagocytosis

**FIGURE 1:**
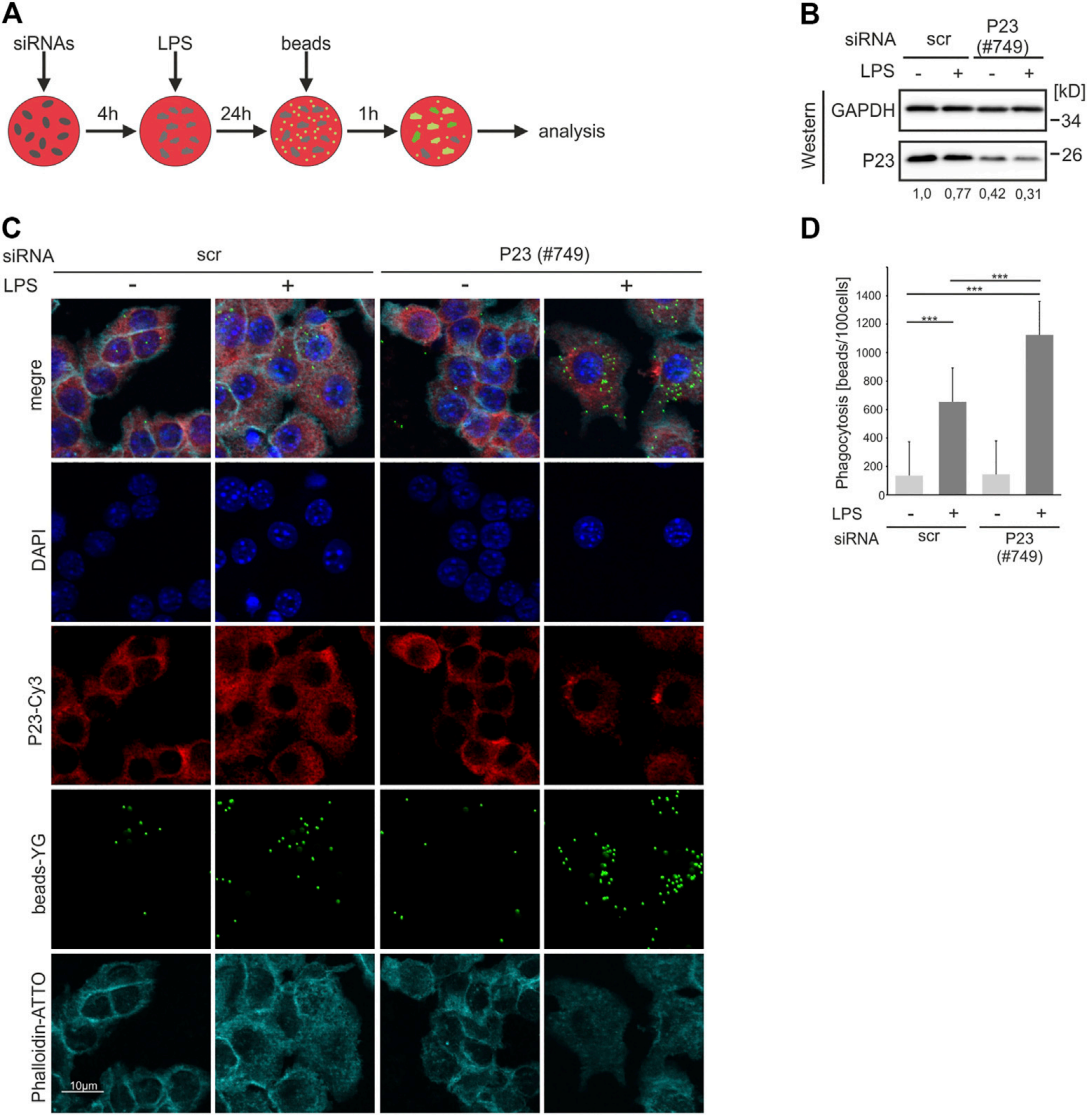
P23 depletion enhances phagocytosis by LPS-induced RAW 264.7 macrophages. **(A)** Schematic representation of the experimental design. **(B)** Cells were transfected with control (scr) or *P23*-specific siRNAs (#749) and stimulated with LPS (100 ng/ml) (*n* = 3). Representative Western blot of cytosolic RAW 264.7 cell extracts with antibodies as indicated, P23 was normalized to GAPDH. **(C)** Following siRNA transfection and LPS treatment, cells were incubated with Fluoresbrite® carboxylate labeled latex beads and analyzed by IF microscopy, as indicated. **(D)** The phagocytosis index was calculated as 10^3^ beads ingested by 100 cells. Statistical analysis was performed with one-way ANOVA, significance levels defined as *** = *p* < 0.001.

Whereas expression of P23, which we identified as a new RBP in RAW 264.7 macrophages is not affected by 2 h LPS treatment, its poly(A)^+^ RNA binding activity is reduced in response to LPS (Liepelt et al., 2016). In order to elucidate P23 RBP functions we asked whether the impact of LPS on RNA binding affects the phagocytotic activity of macrophages, a critical function in pathogen clearance (Stuart and Ezekowitz, 2005). To diminish P23 expression, P23-specific and control siRNAs, were transfected in RAW 264.7 cells 4 h prior to 24 h LPS treatment and labelled latex beads supply for 1 h (Figure 1A). P23 was detected by Western blotting (Figure 1B) and phagocytosed beads were analyzed and quantified by immunofluorescence microscopy (IF) (Figures 1C,D). LPS induced phagocytosis was strongly elevated in P23 depleted macrophages compared to the control (Figures 1C,D). The impact of P23 depletion on macrophage activity along with the declining binding of poly(A)^+^-RNA by P23 in response to LPS (Liepelt et al., 2016) motivated us to identify P23 target mRNAs and to explore if it exhibits functions in the post-transcriptional regulation of gene expression.

### P23 Interacts Specifically With Kinesin 15 mRNA

**FIGURE 2:**
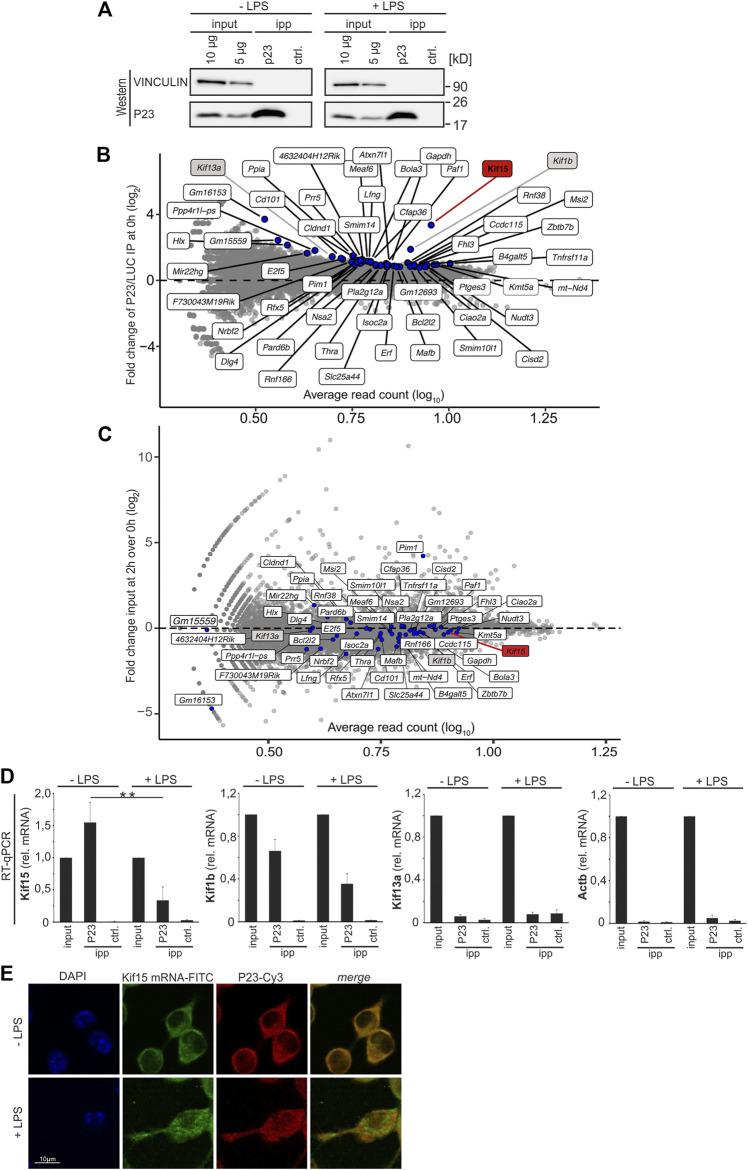
Identification of P23 interacting mRNAs in RAW 264.7 cells. **(A)** P23 immunoprecipitation (P23, antibody JJ3) from cytoplasmic extracts of untreated or LPS-induced RAW 264.7 cells (10 ng/ml, 2 h), which were cross-linked at λ = 254 nm. A LUC antibody served as control (ctrl.) (*n* = 3). **(B)** Specific enrichment of 52 RNAs in the P23 IP compared to LUC IP at the 0 h time point. Significantly enriched RNAs highlighted labeled in blue (adjusted *p*-value ≤ 0.05, Benjamini Hochberg correction, log_2_-transformed fold change >0). **(C)** Expression level of the 52 RNAs in the input of untreated RAW 264.7 cells and at 2 h LPS treatment. **(D)** Analysis of *Kif15, Kif1b and Kif13a* mRNAs enrichment by qPCR, *Actb* mRNA served as control, normalized to exogenously added Luc mRNA. **(E)** RAW 264.7 cells either left untreated or induced with LPS were hybridized with a *Kif15* mRNA probe (FITC, green) for *in situ* hybridization. Immunostaining of endogenous P23 (Cy3, red) with a specific antibody, staining of nuclei with DAPI. Statistical analysis was performed with one-way ANOVA, significance levels defined as ** = *p* < 0.01.

Initially we set out to identify mRNAs that were differentially bound by P23 in untreated and LPS-induced macrophages. P23 was immunoprecipitated (IP) from cytoplasmic extracts of UV-cross-linked RAW 264.7 untreated macrophages (-LPS) and after 2 h LPS treatment (+LPS). A luciferase antibody served as control (ctrl.) for both conditions (Figure 2A). RNA sequencing (RNAseq) identified 52 RNAs as specifically enriched with P23 compared to the LUC IP at the 0 h time point (adjusted *p*-value ≤ 0.05, Benjamini Hochberg correction, log_2_-transformed fold change >0) (Figure 2B). Of those 44 mRNAs possessed Ensembl genome database entries (Table 1). In response to LPS their enrichment was reduced. The majority of encoded proteins represent transcription factors, cytoskeletal proteins and signaling factors (PANTHER) (Thomas et al., 2003; Mi et al., 2013) (Table 1, Supplementary Table S1). Considering the relevance of cytoskeletal remodeling for macrophage migration and phagocytosis (Harrison and Grinstein, 2002; Rougerie et al., 2013), kinesin mRNAs encoding cytoskeletal proteins KIF15, KIF1B and KIF13A, were selected for functional analysis. All three mRNAs were equally expressed in untreated and 2 h LPS-induced macrophages employed for RIP analysis (Figure 2C). There binding specificity was validated by P23 IP from cytoplasmic extracts of untreated and LPS-induced macrophages (Figure 2D). Importantly, specific enrichment of *Kif15* mRNA from extracts of untreated cells was significantly diminished in extracts of LPS-induced cells (Figure 2D). Enrichment of *Kif1b* mRNA was less pronounced and *Kif13a* mRNA did not exhibit specific interaction with P23. These results are consistent with the RNAseq analysis demonstrating the most distinct specific binding for *Kif15* mRNA (Table 1).

**TABLE 1:** List of 44 P23 interacting mRNAs, which were differentially enriched in response to LPS.

Symbol	Ensembl	PANTHER protein class	P23/LUC [TPMm_ean_]	
			0 h	2 h
ATXN7L1	ENSMUSG00000020564		2.44	0.99
B4GALT5	ENSMUSG00000017929		2.38	1.23
BCL2L2	ENSMUSG00000089682	Signaling molecule	2.10	1.07
BOLA3	ENSMUSG00000045160		2.70	1.06
CCDC115	ENSMUSG00000042111		2.32	0.97
CD101	ENSMUSG00000086564		3.11	0.98
CFAP36	ENSMUSG00000020462		2.54	0.91
CISD2	ENSMUSG00000028165		2.04	1.05
CLDND1	ENSMUSG00000022744		2.77	1.14
DLG4	ENSMUSG00000020886	Transmembrane receptor	2.42	1.56
E2F5	ENSMUSG00000027552	Transcription factor	2.88	0.44
ERF	ENSMUSG00000040857	Signaling molecule, transcription factor	2.19	1.05
FAM96A	ENSMUSG00000032381		2.09	1.01
FHL3	ENSMUSG00000032643	Transcription factor	2.42	1.17
GAPDH	ENSMUSG00000057666	Oxidoreductase	2.25	1.02
HLX	ENSMUSG00000039377	Transcription factor	4.72	1.28
ISOC2A	ENSMUSG00000086784		2.26	1.31
KIF13A	ENSMUSG00000021375	Cytoskeletal protein	3.33	0.73
KIF15	ENSMUSG00000036768	Cytoskeletal protein	12.01	7.80
KIF1B	ENSMUSG00000063077	Cytoskeletal protein	4.35	2.20
KMT5A	ENSMUSG00000049327		2.32	1.09
LFNG	ENSMUSG00000029570	Transferase, signaling molecule	2.76	0.96
MAFB	ENSMUSG00000074622	Transcription factor	2.13	1.21
MEAF6	ENSMUSG00000028863		2.70	1.38
MSI2	ENSMUSG00000069769		2.19	1.08
NRBF2	ENSMUSG00000075000		2.63	0.97
NSA2	ENSMUSG00000060739		2.28	0.87
NUDT3	ENSMUSG00000024213	Hydrolase, phosphatase	2.17	1.19
PAF1	ENSMUSG00000003437		2.33	1.07
PARD6A	ENSMUSG00000044641	Cell junction protein (tight junction)	2.48	0.92
PIM1	ENSMUSG00000024014	Kinase, transferase, receptor	2.71	0.97
PLA2G12A	ENSMUSG00000027999		2.27	1.13
PPIA	ENSMUSG00000071866	Isomerase	3.62	1.13
PRR5	ENSMUSG00000036106		2.80	1.10
PTGES3	ENSMUSG00000071072	Chaperone	2.14	1.16
RFX5	ENSMUSG00000005774	Transcription factor	2.25	1.05
RNF166	ENSMUSG00000014470	Ligase	2.25	1.07
RNF38	ENSMUSG00000035696		2.54	0.90
SLC25A44	ENSMUSG00000050144	Carrier protein, transporter, calcium binding	2.03	0.82
SMIM10L1	ENSMUSG00000072704		2.03	1.04
SMIM14	ENSMUSG00000037822		2.66	1.24
THRA	ENSMUSG00000058756	Receptor, transcription factor	2.48	1.13
TNFRSF11A	ENSMUSG00000026321	Receptor	2.52	1.26
ZBTB7B	ENSMUSG00000028042	Nucleic acid binding	2.29	0.90

Moreover, immunofluorescence and fluorescence *in situ* hybridization (IF-FISH) demonstrated an LPS-dependent decline in P23-*Kif15* mRNA co-localization (Figure 2E), consistent with a reduced P23-poly(A)^+^ RNA interaction (Liepelt et al., 2016). These results initially indicated that the modulation of the P23-*Kif15* mRNA might contribute to macrophage functional control.

**FIGURE 3:**
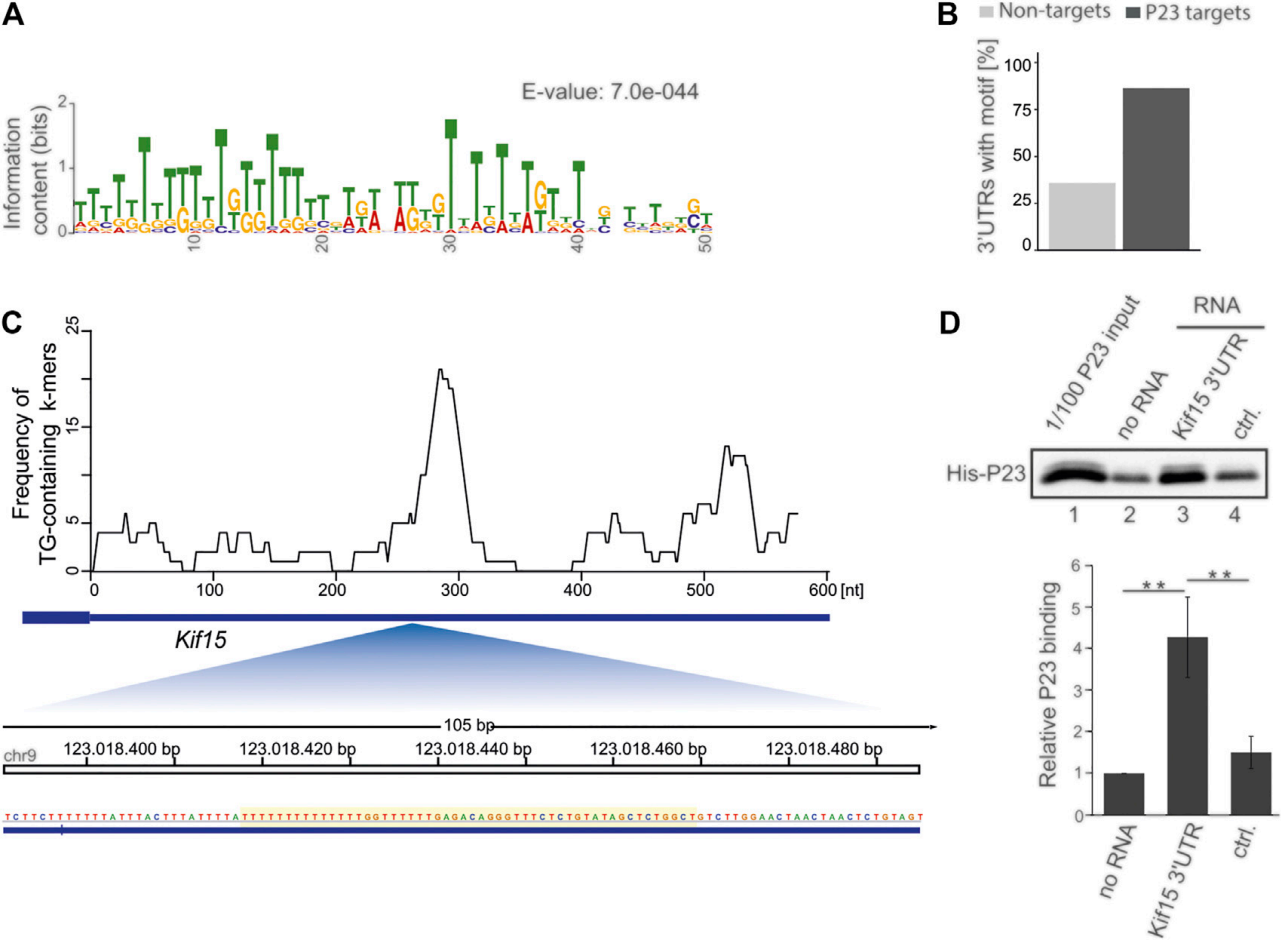
Discriminative motif enrichment analysis in P23 target mRNA 3’UTRs. **(A)** The sequence logo represents the information content (column high) and relative frequency of each nucleotide (relative height within the column) at each position of the motif. **(B)** Frequency of the U/G-rich motif identified by MEME in 44 P23 target mRNAs vs. 117 unbound mRNAs, which were equally expressed but did not exhibit P23 interaction. **(C)** Genome browser view of the *Kif15* 3’UTR (606 nt, NM_010620.1) with frequency profile for U/G-only hexamers (top) and zoom-in on the region harbouring the UG-rich motif identified by MEME (bottom). **(D)** 3’end biotinylated RNAs, *Kif15* mRNA 3’UTR or ALOX15 mRNA 3’UTR DICE (ctrl.), were immobilized on streptavidin beads and incubated with His-P23 (*n* = 3). Following elution, P23 was detected by a specific antibody. Statistical analysis was performed with one-way ANOVA, significance levels defined as ** = *p* < 0.01.

To discern common sequence features of the 44 differentially P23 interacting mRNAs, discriminative motif enrichment analysis was performed for the 5′UTR, 3′UTR and open reading frame sequences (Bailey and Elkan, 1994; Bailey et al., 2009) (Figure 3). The search identified a U/G-rich motif consisting of U/G hexamers (E-value: 7.0e-044) (Figure 3A) in the 3’UTR of 38 mRNAs (86%), while a similar 3′UTR motif appeared in less than 30% of unbound control mRNAs (Figure 3B, Supplementary Table S1). The motif search in the 5′UTR and ORF of the 44 P23 target mRNAs did not retrieve meaningful motifs (data not shown). We investigated RNA folds in the vicinity of the identified U/G-rich motifs that could potentially be involved in P23 binding, but due to the low number of sequences, we cannot draw reliable conclusions whether any such Minimal Free Energy (MFE) fold could potentially be involved in P23 binding (data not shown). The position of all detected motif instances in the 3′UTRs of those P23 target mRNAs with an *p*-value with less than 0.0001 is shown in the Supplementary Figure S1. Consistent with the P23 IP, a distinct U/G motif mapped to the *Kif15* mRNA 3′UTR (E-value 6.7e^−18^, *p*-value 7.74e^−21^) (Figure 3C, Supplementary Table S1), whereas it aligned to less extent with the *Kif1b* mRNA 3′UTR (E-value 7.5e^−5^, *p*-value 4.54e^−9^) and was absent from *Kif13a* mRNA (E-value 5.8e^+0^) (Supplementary Table S1).

In order to examine specific P23 binding to the U/G motif *in vitro*, we made use of recombinant P23 and 3′biotinylated transcripts coupled to streptavidin beads (Figure 3D). As control we employed the well characterized ALOX15 mRNA 3’UTR U/C-repeat sequence (DICE) that is devoid of U/Gs, confers HNRNPK binding and mediates translational regulation (Ostareck-Lederer et al., 1994; Ostareck et al., 1997; Moritz et al., 2014). His-P23 strongly bound to U/G motif bearing *Kif15* mRNA 3′UTR (Figure 3D, lane 3), whereas no binding exceeding the empty beads background was detected for the DICE control (Figure 3D, lanes 2 and 4). These results indicate that the U/G motif constitutes a P23 binding scaffold.

### *Kif15* mRNA and Protein Are Diminished in Lipopolysaccharides Activated Macrophages

**FIGURE 4:**
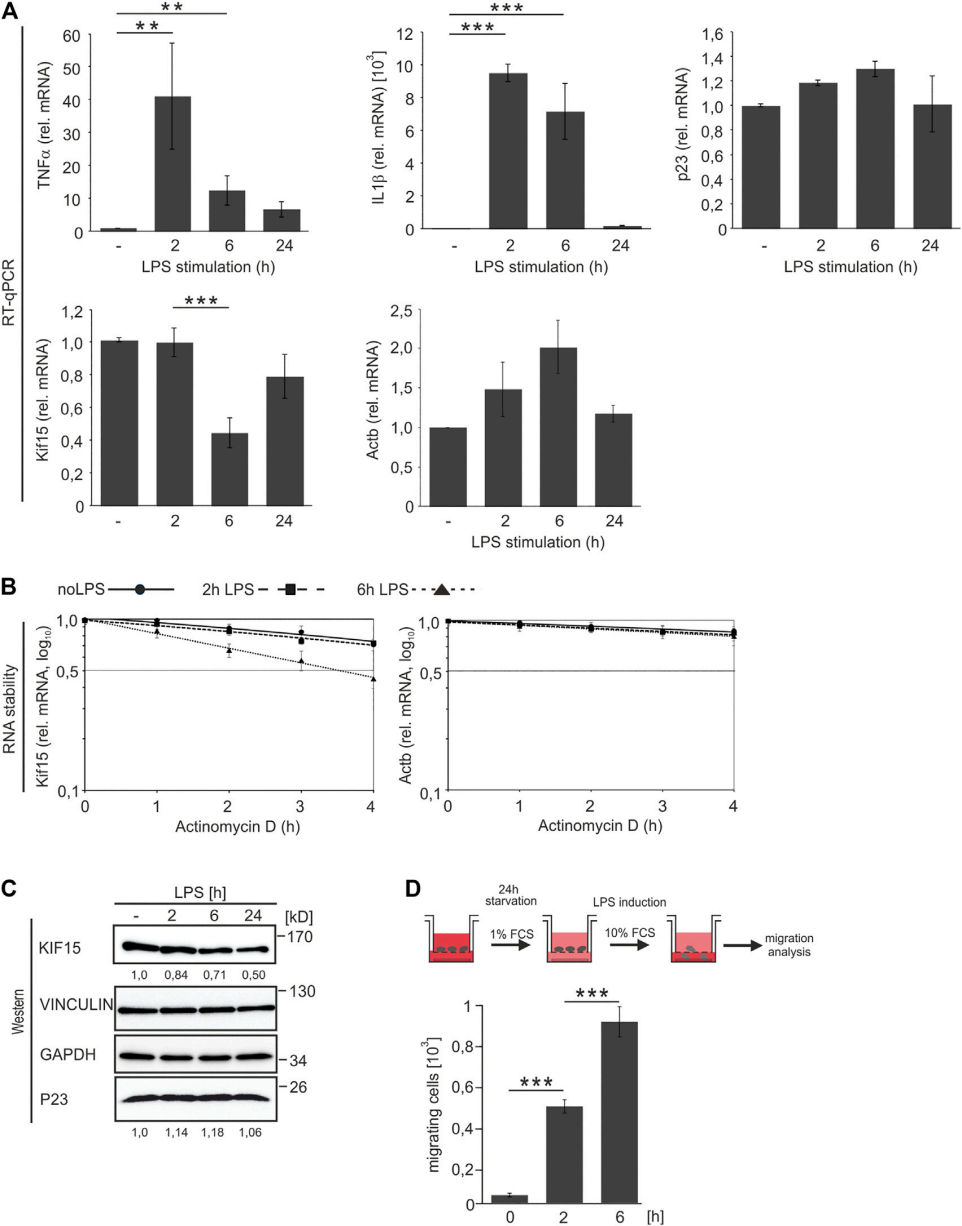
P23 and KIF15 expression in untreated and LPS-induced macrophages. LPS induction of RAW 264.7 macrophages (10 ng/ml, 2, 6 and 24 h) (*n* = 4). **(A)** QPCR analysis of mRNA levels as indicated, normalized to *Ndufv1* mRNA (*n* = 4). **(B)** Inhibition of transcription in untreated cells and after 2 and 6 h LPS induction as indicated (*n* = 3). *Kif15* and *Actb* mRNA were monitored by qPCR, normalized to exogenously added Luc mRNA. **(C)** Representative Western blot with specific antibodies as indicated, KIF15 was normalized to VINCULIN and P23 to GAPDH. **(D)** Upper panel: Schematic representation of the experimental design. Lower panel: RAW 264.7 cell migration in response to LPS (*n* = 3). Statistical analysis was performed with one-way ANOVA, significance levels defined as ** = *p* < 0.01 and *** = *p* < 0.001.

The impact of LPS on the expression of P23 and KIF15 was addressed in time course analyses of both mRNA and protein expression (Figure 4). Efficient RAW 264.7 macrophage activation over 24 h was clearly reflected in the expression kinetics of cytokine mRNAs TNFα and IL1β, which were sharply increased after 2 h and subsequently declined, distinctive for LPS-driven TLR4 signaling (Figure 4A; Liepelt et al., 2014). An increase of P23 mRNA until 6 h followed by a slight reduction (Figure 4A), was also observed at the protein level (Figure 4C). Interestingly, *Kif15* mRNA exhibited a sharp drop after 6 h that was reversed until 24 h (Figure 4A), in contrast to continuously declining KIF15 protein, whereas GAPDH and VINCULIN remained unaffected (Figure 4C). *Actb* mRNA did not change significantly (Figure 4A). The stability of *Kif15* mRNA was only slightly diminished in both untreated and 2 h LPS-induced cells, however 6 h LPS treatment resulted in a distinct destabilization (t_1/2=_3.5 h) (Figure 4B, left panel), concurring with *Kif15* mRNA reduction at that time point (Figure 4A). *Actb* mRNA was only slightly affected (Figure 4B, right panel). A comparable *Kif15* mRNA half-life (t_1/2_ = 5–6 h) was observed in differentiating mouse embryonic stem cells (Sharova et al., 2009). KIF15, also termed Kinesin-12, was originally described as motor protein implicated in mitotic spindle formation (Kashina et al., 1996; Scholey, 2009; Ferenz et al., 2010). Recent studies in neurons indicate functions of KIF15 in the regulation of cell morphology, axonal growth and branching (Buster et al., 2003; Liu et al., 2010), as well as dendrite differentiation and neuronal migration (Lin et al., 2012; Drechsler and Mcainsh, 2016; Feng et al., 2016).

To examine whether LPS-induced macrophage migration (Tajima et al., 2008) is linked to declining KIF15, we established a RAW 264.7 migration assay (Figure 4D). Clearly, enhanced macrophage migration was significantly progressing until 6 h post LPS addition (Figure 4D), when *Kif15* mRNA stability and abundance declined (Figures 4A,B). These observations suggest that LPS entails alterations in *Kif15* mRNA stability, which enable a dynamic macrophage migration response. These results are in agreement with the observed increased movement of KIF15-depleted astrocytes (Feng et al., 2016).

### P23 Depletion Destabilizes *Kif15* mRNA and Activates Migration of Untreated Macrophages

**FIGURE 5:**
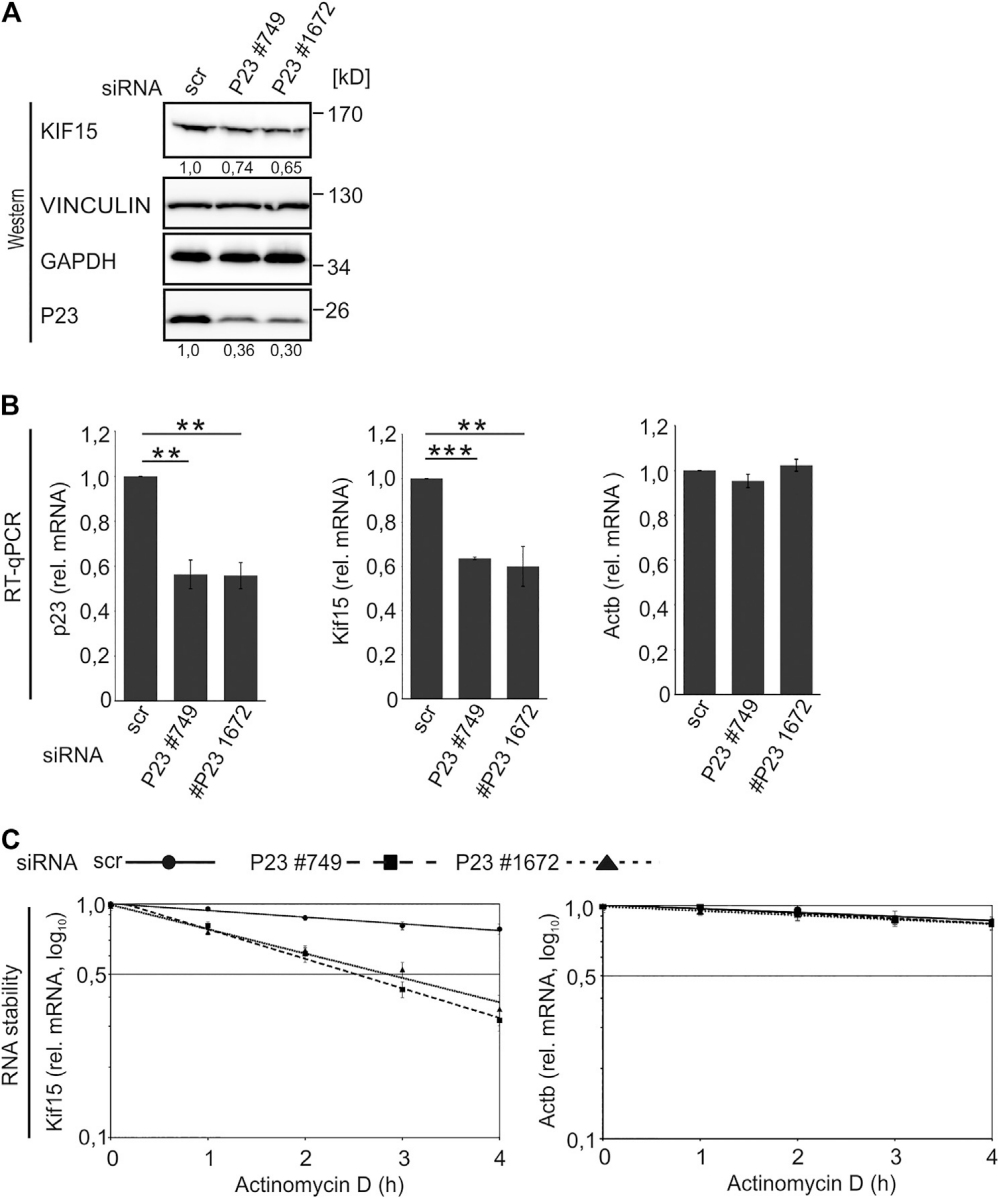
P23 depletion from untreated macrophages results in *Kif15* mRNA and protein decline. **(A)** RAW 264.7 cells were transfected with control (scr) or *P23* siRNAs (#749, #1672) (*n* = 3). Representative Western blot with antibodies as indicated. KIF15 was normalized to VINCULIN and P23 to GAPDH. **(B)** Analysis of mRNA levels by qPCR, normalized to *Ndufv1* mRNA (*n* = 3). **(C)** Inhibition of transcription in untreated cells, as indicated (*n* = 3). *Kif15* and *Actb* mRNAs were monitored by qPCR, normalized to exogenously added Luc mRNA. Statistical analysis was performed with one-way ANOVA, significance levels defined as ** = *p* < 0.01 and *** = *p* < 0.001.

The interaction of P23 with *Kif15* mRNA in untreated macrophages, which strongly declined following LPS treatment (Table 1; Figures 2D,E), pointed us to a potential impact of P23 on *Kif15* mRNA stability. To address this, P23 was depleted from untreated RAW 264.7 macrophages employing two specific siRNAs (#749, #1672) (Figure 5). Interestingly, KIF15 protein and *Kif15* mRNA were specifically reduced as a consequence of diminished P23 expression (Figures 5A,B). Furthermore, *Kif15* mRNA was destabilized when P23 expression was diminished (Figure 5C), indicating a stabilization by P23 in untreated macrophages. A combination of P23 depletion with LPS treatment did not result in further KIF15 decline (data not shown), probably due to a reduced P23-*Kif15* mRNA interaction after 2 h (Table 1; Figures 2D,E).

**FIGURE 6:**
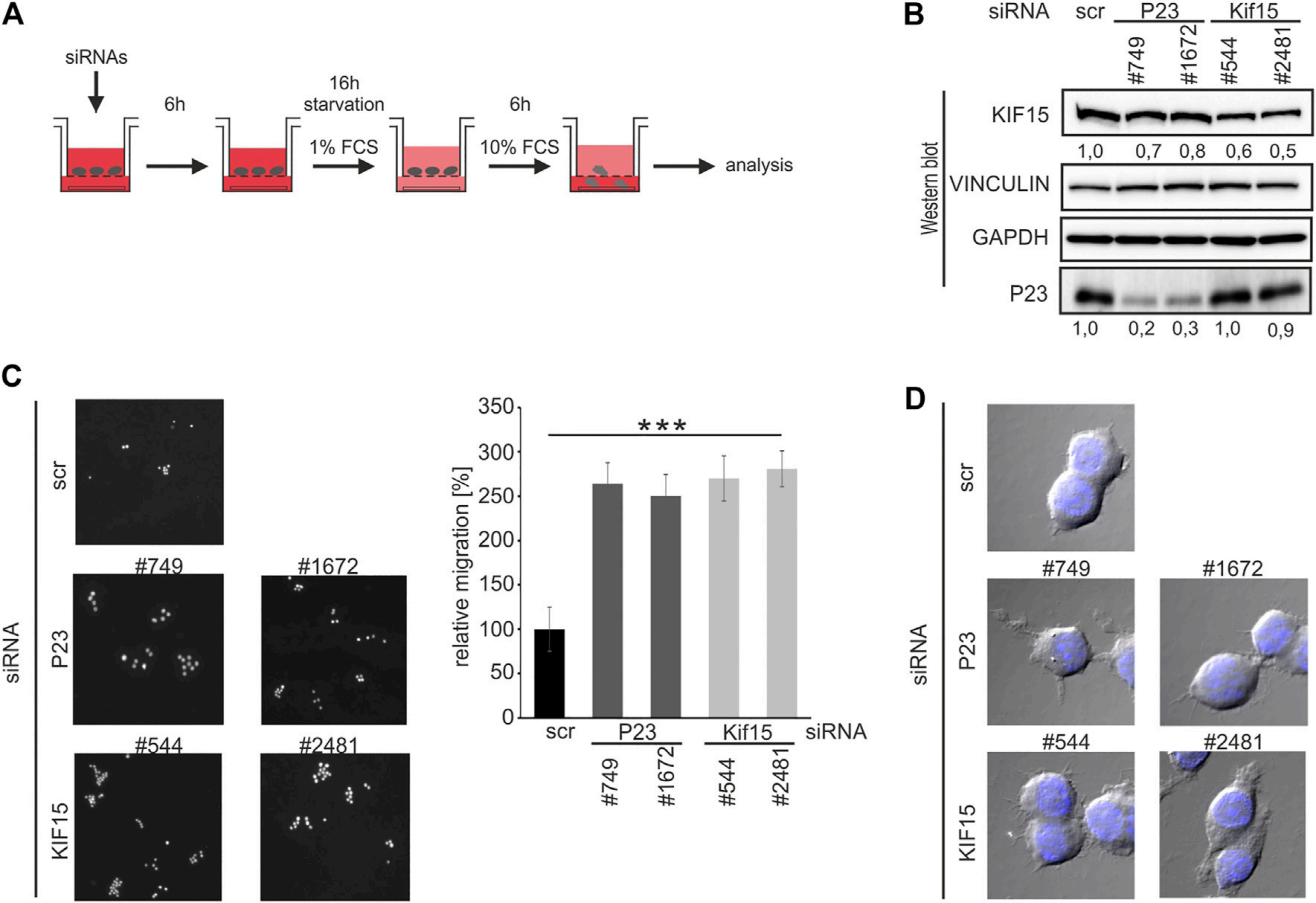
P23 or KIF15 depletion enhances untreated macrophage migration. **(A)** Schematic representation of the experimental design. RAW 264.7 cells were transfected with control (scr), *P23* (#749, #1672) or *Kif15* (#544, #2481) specific siRNAs and treated as depicted (*n* = 3). **(B)** Representative Western blot with antibodies as indicated. KIF15 was normalized to VINCULIN and P23 to GAPDH. **(C)** Left panel: Analysis of migrating cells by microscopy. Right panel: Quantification of migrating cells (n = 3). **(D)** Morphology of migrated cells (DAPI staining and bright-field microscopy). Statistical analysis was performed with one-way ANOVA, significance levels defined as *** = *p* < 0.001.

Since declining KIF15 correlated with enforced macrophage migration (Figures 4A,C,D), we integrated P23 as well as KIF15 depletion in a migration assay (Figure 6A). Attenuation of P23 and KIF15 (siRNAs #544, #2481) led to declining KIF15 expression in untreated macrophages (Figure 6B). Noteworthy, both P23 depletion by siRNAs, as well as the direct siRNA-mediated KIF15 reduction, led to a significantly elevated migration activity (Figure 6C). Moreover, augmented pseudopodia formation of siRNA-transfected cells corresponds with enhanced macrophage migration (Figure 6D). Again, a combination of P23 depletion with LPS treatment did not result in further KIF15 decline (data not shown).

The finding that P23 depletion attenuates *Kif15* mRNA stability and protein expression (Figures 5A,B,C, 6B) and furthermore drives untreated macrophage migration (Figure 6C), supports the hypothesis that the dynamic formation of P23-*Kif15* mRNA ribonucleoprotein complexes (mRNPs) contributes to macrophage mobility control.

### P23 Interacts With Casein Kinase 2 and Lipopolysaccharides-dependent With HSP90

**FIGURE 7:**
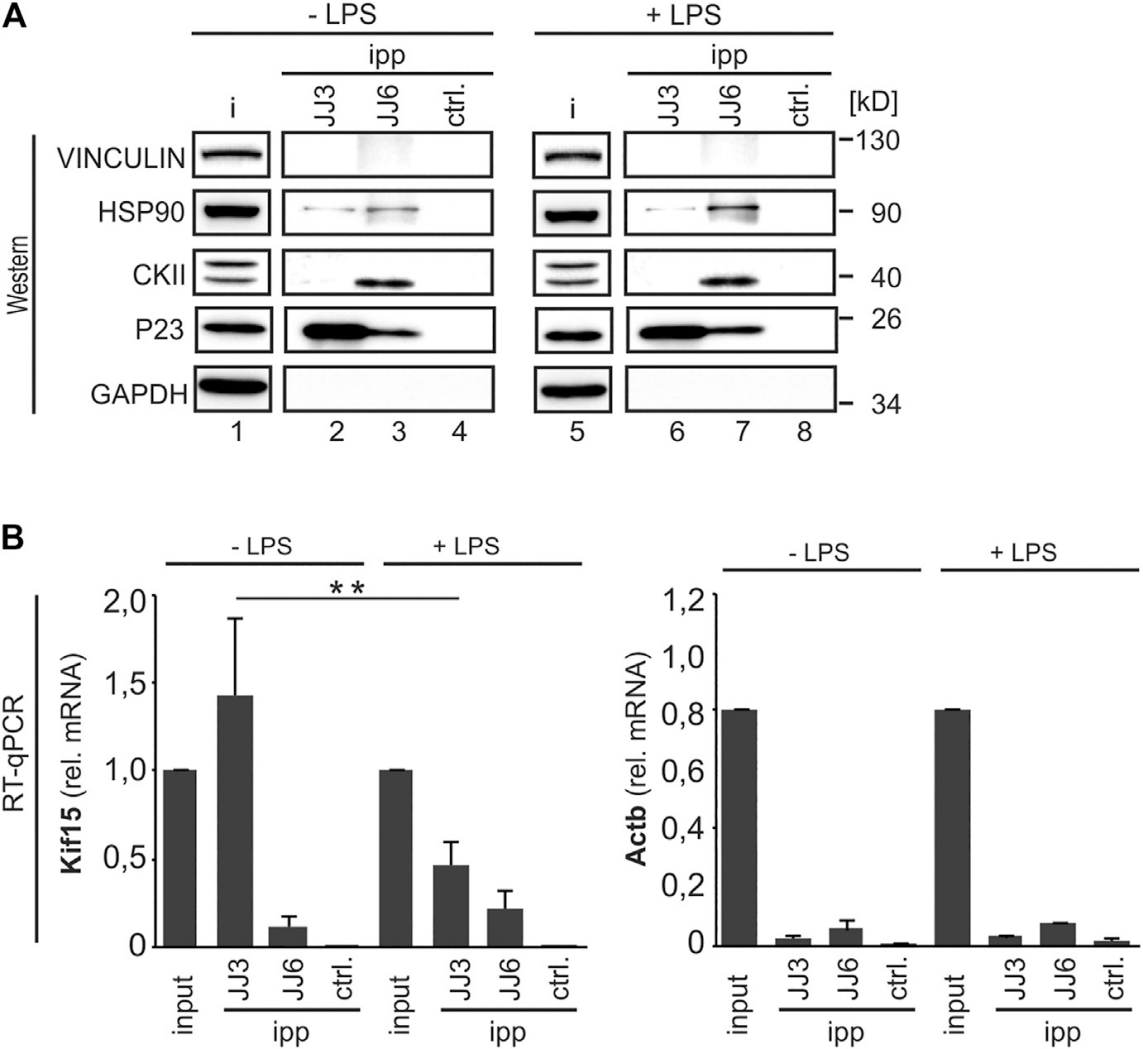
P23 interacts with Casein kinase 2 and LPS-dependent with HSP90. **(A)** P23 immunoprecipitation (JJ3 or JJ6) from cytoplasmic extracts of untreated (−LPS) or LPS-induced (+LPS) RAW 264.7 cells (10 ng/ml, 2 h), which were cross-linked at λ = 254 nm. A LUC antibody served as control (ctrl.) (*n* = 3). Representative Western blot with antibodies as indicated. **(B)** *Kif15* and *Actb* mRNAs were monitored by qPCR, normalized to exogenously added Luc mRNA (*n* = 3). Statistical analysis was performed with one-way ANOVA, significance levels defined as ** = *p* < 0.01.

Besides its function as HSP90 co-chaperone, P23 possesses cytosolic prostaglandin E synthase activity, converting COX-1, but not COX-2-derived prostaglandin PGH2 to PGE2 (Tanioka et al., 2000). In activated cells, casein kinase (CK)2-dependent P23 phosphorylation is linked to increased enzymatic activity and PGE2 production, both facilitated by HSP90 interaction. HSP90 may act as a scaffold that brings P23 and CK2 in close proximity within the P23-CK2-HSP90 complex (Tanioka et al., 2003; Kobayashi et al., 2004; Hara et al., 2010). To address the potential interaction we immunoprecipitated P23 from cytoplasmic extracts of UV-cross-linked untreated RAW 264.7 cells (−LPS) and after 2 h LPS induction (+LPS) with two different P23 antibodies (JJ3 and JJ6, generated against full-length recombinant P23). A luciferase antibody served as control (ctrl.) (Figure 7A). Interestingly, JJ6 immunoprecipitated CK2 and HSP90, the latter more effective from LPS treated cells, but did not co-precipitate the *Kif15* mRNA. In contrast, efficiently *Kif15* mRNA precipitation was demonstrated from untreated cells, which declined after LPS treatment, employing JJ3. However, JJ3 did not co-precipitate CK2 and HSP90. (Figures 7A,B). This indicates that P23 acts as an RBP in untreated cells, while the interaction with HSP90 increases in LPS-stimulated cells. Furthermore, the results may suggest that different structural determinants confer P23-*Kif15* mRNA interaction and binding of CK2 and HSP90.

## Discussion

Besides its function as a HSP90 co-chaperone (Felts and Toft, 2003), it was shown that P23 possesses cytosolic PTGES3 activity (Tanioka et al., 2000), which is elevated in response to LPS in peritoneal macrophages (Naraba et al., 1998). We identified P23 as a novel RBP that exhibits differential poly(A)^+^ RNA binding in untreated macrophages and cells activated in response to LPS (Liepelt et al., 2016), with so far unknown implications for macrophage function. Interestingly, P23 depletion caused an increase in the phagocytic activity of LPS-induced RAW 264.7 macrophages (Figure 1). In context of the differential poly(A)^+^ RNA interaction (Liepelt et al., 2016) this finding motivated us to explore a potential post-transcriptional impact on specific mRNAs. P23 target mRNA fractions immunoprecipitated from cytoplasmic extracts of cross-linked untreated and LPS activated RAW 264.7 cells (RIP) were analyzed by RNAseq and examined for differential P23 association in untreated and activated macrophages (Figure 2). Annotation of proteins encoded by the identified 44 mRNAs revealed an over-representation of transcription factors, cytoskeletal components and proteins involved in signal transduction (Table 1, Supplementary Table S1).

Cytoskeletal proteins are vitally important for macrophage migration and phagocytosis, both connected with extensive cytoskeletal remodeling (Rougerie et al., 2013). The motor protein KIF15 was originally described to participate in spindle formation (Kashina et al., 1996; Scholey, 2009; Ferenz et al., 2010). A diminished interaction of P23 with *Kif15* mRNA in response to LPS is evident from co-precipitation and co-localization analyses (Figures 2D,E). The *Kif15* mRNA decline between 2 and 6 h LPS treatment (Figures 4A,B) suggests that in activated macrophages the loss of P23 association triggers the decay of *Kif15* mRNA. The reduced amount of *Kif15* mRNA that is accompanied by a diminished *Kif15* mRNA stability in untreated macrophages, from which P23 is depleted (Figures 5A,B,C), supports this hypothesis. Furthermore, after 2 h LPS treatment P23 binding to *Kif15* mRNA is significantly reduced (Figure 2D), which could explain why LPS treatment combined with P23 depletion does not cause further KIF15 decline (data not shown).

Discriminative motif enrichment analysis revealed that U/G motifs are specifically enriched in the 3′UTR of P23 target mRNAs, particularly the *Kif15* mRNA 3′UTR (Figures 3A,B,C, Supplementary Table S1), in contrast to unbound mRNAs. The importance of these U/G motifs for specific P23 binding became apparent in *vitro* RNA-interaction assays where recombinant P23 was bound by the *Kif15* mRNA 3′UTR, but not a control RNA consisting of U/C repeats, devoid of U/G motifs (Figure 3D).

Not only for the HSP90 co-chaperone P23 functional poly(A)^+^ RNA interaction was demonstrated (Liepelt et al., 2016), but also for HSPBP1 a HSP70 co-chaperone, which features an *in vitro* preference for pyrimidine-rich sequences (Mahboubi et al., 2020). Specifically, HSPBP1 is implicated in stress-granule formation control and interacts with related proteins G3BP1, HUR and TIA-1/TIAR (Mahboubi et al., 2020). In addition, the chaperone HSP70 independent of its function, exhibits *in vitro* affinity for AU-rich elements (ARE) and stabilizes the ARE containing *VEGFA* mRNA *in vivo* (Kishor et al., 2017).

Interestingly, a motor protein is encoded by *Kif15* mRNA, which is differentially bound by P23 in untreated and LPS-activated macrophages. KIF15 depletion was shown to drive accelerated migration of rat cortical astrocytes (Feng et al., 2016) to contribute to decreased cell proliferation and an extended neuronal axon outgrowth in rodents (Liu et al., 2010). Furthermore, KIF15 mutations accelerate the axonal outgrowth during zebrafish neuronal development and regeneration (Dong et al., 2019). In accordance with the finding that KIF15 diminished conditions lead to an increase in microtubule mobility, these studies point to a function of KIF15 as a brake acting in microtubule transport (Kahn and Baas, 2016; Rao and Baas, 2018; Dong et al., 2019).

Consistently, with decreasing *Kif15* mRNA and protein level (Figures 4A,C) we observed an enhanced migration of activated RAW 264.7 macrophages (Figure 4D) Therefore we hypothesized that the P23-*Kif15* mRNP might contribute to macrophage migration control. P23 or KIF15 depletion from RAW 264.7 cells not exposed to LPS boosted macrophage migration (Figure 6C) and the formation of podosomal protrusions (Figure 6D). Similarly HSP70/90-organizing protein HOP/STI1 was found to contribute to neuroblast migration control (Miyakoshi et al., 2017).

Considering the prominent impact of macrophages on innate immune response control, it would be interesting to search for small molecules, which stabilize the P23-*Kif15* mRNA interaction to inhibit macrophage migration as recently suggested (Gomez-Rial et al., 2020). Alternatively, the innate macrophages immune response could be primed (Deng et al., 2013) through P23-*Kif15* mRNA interaction inhibitors.

Noteworthy, besides it co-chaperone function P23 features cytosolic PTGES3 activity, as demonstrated in LPS-activated peritoneal macrophages (Naraba et al., 1998). Enabled through the interaction with HSP90, CK2-catalyzed P23 phosphorylation leads to elevated enzymatic activity and PGE2 production (Tanioka et al., 2003; Kobayashi et al., 2004; Hara et al., 2010).

P23 immunoprecipitation with two different antibodies, revealed that P23 acts as an RBP in untreated RAW 264.7 macrophages (Figures 2,7). In response to LPS its interaction with HSP90, but not CK2 is enhanced (Figure 7), consistently induction of PTGES3 activity was accompanied by an increase in PTGES3-HSP90 complex formation (Tanioka et al., 2003). In addition, our immunoprecipitation studies suggest that the *Kif15* mRNA interaction and binding of CK2 and HSP90 are mediated by different structural determinants. P23 consists of two modules, a compact β-strand CS domain (CHORD-containing proteins and SGT1) providing a HSP90 binding surface and an unstructured C-terminal tail that is necessary for optimal chaperone activity (Weikl et al., 1999; Weaver et al., 2000). For disordered protein regions a RNA chaperone function has been described (Semrad, 2011) and it was shown that the unstructured C-terminal tail of HIV-1 VIF confers RNA binding (Sleiman et al., 2014).

To investigate a possible contribution of P23/PTGES3 to PGE2 production in LPS-treated RAW264.7 cells, we analyzed culture supernatants of untreated and P23-depleted cells in response to LPS (data not shown). The only moderate reduction of PGE2 production, which was determined could be explained by the activity of two additional PGE synthetases besides PTGES3: inducible microsomal mPGES-1 and the constitutively expressed PGES-2 (Jakobsson et al., 1999; Murakami et al., 2000; Tanikawa et al., 2002). While mPGES-1 is functionally coupled to inducible COX2, mPGES-2 functions equally well with both COX-1 and COX-2 (Hara et al., 2010). To answer this question, an in depth enzymatic analysis of the contribution of individual PGE synthetases in macrophages would be required.

Furthermore, P23 HSP90 co-chaperone function could also be affected by LPS as HSP90 activity is essential for the stabilization and maturation of proteins involved in NFκB activation and inflammation (Chatterjee et al., 2007; Chatterjee et al., 2008; Thangjam et al., 2014). Also in this context, the catalytic activity of CK2 contributes to the modulation of HSP90 chaperone activity (Miyata, 2009).

**FIGURE 8:**
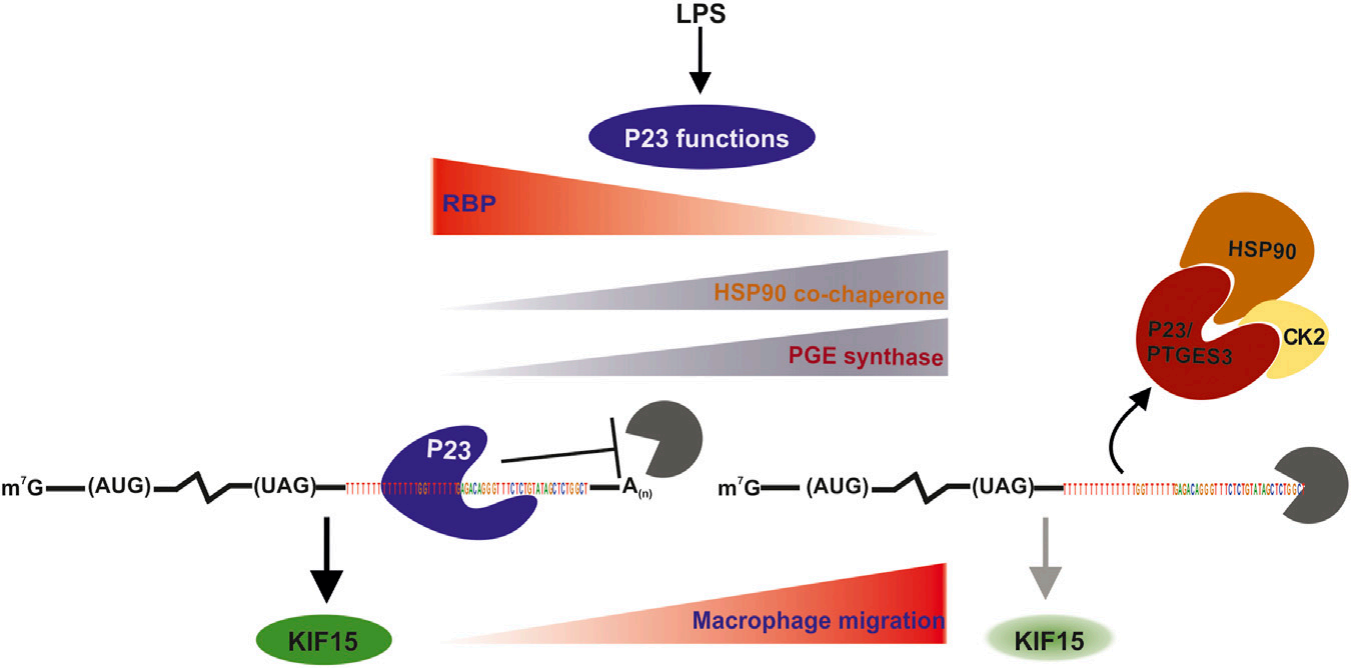
Model of *Kif15* mRNA stability regulation by P23. In untreated macrophages P23 bound to 3’UTR U/G motifs stabilizes *Kif15* mRNA. Newly synthesized KIF15 protein suppresses macrophage migration. Macrophage LPS induction leads to P23 release and *Kif15* mRNA destabilization. Diminished KIF15 expression results in increased macrophage migration, as previously reported for rat cortical astrocytes (Feng et al., 2016). P23 exhibits cytosolic PTGES3 activity, which was demonstrated in LPS-activated peritoneal macrophages (Naraba et al., 1998). Facilitated by HSP90 interaction CK2-catalyzed PTGES3 phosphorylation is linked to increased enzymatic activity and PGE2 production (Tanioka et al., 2003; Kobayashi et al., 2004; Hara et al., 2010). In addition, P23 could also act LPS-dependent as HSP90 co-chaperone, since HSP90 activity is essential for the stabilization and maturation of proteins involved in NFκB activation and inflammation (Chatterjee et al., 2007; Chatterjee et al., 2008; Thangjam et al., 2014). Also in this context, the catalytic activity of CK2 contributes to the modulation of HSP90 chaperone activity (Miyata, 2009).

Our new findings related to P23 as an RBP are summarized in Figure 8 in the context of its known cellular activities. From our data it is conceivable that the function of P23 as an RBP in untreated macrophages is superseded by its enzymatic activity or HSP90 co-chaperone function in LPS activated macrophages, where its RNA binding activity declines. Future studies will focus on the minimal RNA sequence features required for P23 binding, the identification of its RNA-interaction domain and RNA-binding control.

## Data Availability

The datasets presented in this study can be found in online repositories. The names of the repository/repositories and accession number(s) can be found below: https://www.ncbi.nlm.nih.gov/, GSE159619.

## Abbreviations

ALOX15, arachidonate-15-lipoxygenase; ATP, adenosine triphosphate; CK2, casein kinase two; FCS, fetal calf serum; G3BP1, Ras GTPase-activating protein-binding protein one; HNRNPK, heterogeneous ribonucleoprotein K; HOP/STI1, HSP70/HSP90 Heat shock organizing protein/stress inducible phosphoprotein one; HSP, heat shock protein; HSPBP1, heat shock protein 70 binding protein one; HUR, human antigen R; KIF15, kinesin family member 15; LPS, lipopolysaccharides; MAPK, mitogen activated kinase; mRNP, messenger ribonucleoprotein complex; NFκB, nuclear factor kappa B; PGE2, prostaglandin E2; PTGES3, prostaglandin E synthase three; qPCR, quantitative polymerase chain reaction; RBP, RNA binding protein; RNAi, RNA interference; RNAseq, RNA sequencing; siRNA, small interfering RNA; TIAR, T-cell intracellular antigen one related protein; TLR4, toll-like receptor four; TTP, tristetraprolin; VEGF, vascular endothelial growth factor.

## References

[B1] Anders S. Pyl P. T. Huber W. (2015). HTSeq–a Python Framework to Work with High-Throughput Sequencing Data. Bioinformatics 31, 166–169. 10.1093/bioinformatics/btu638 25260700 PMC4287950

[B2] Bailey T. L. Elkan C. (1994). Fitting a Mixture Model by Expectation Maximization to Discover Motifs in Biopolymers. Proc. Int. Conf. Intell. Syst. Mol. Biol. 2, 28–36. 7584402

[B3] Bailey T. L. Boden M. Buske F. A. Frith M. Grant C. E. Clementi L. (2009). MEME SUITE: Tools for Motif Discovery and Searching. Nucleic Acids Res. 37, W202–W208. 10.1093/nar/gkp335 19458158 PMC2703892

[B4] Bailey T. L. Gribskov M. (1998). Combining Evidence Using P-Values: Application to Sequence Homology Searches. Bioinformatics 14, 48–54. 10.1093/bioinformatics/14.1.48 9520501

[B5] Baltz A. G. Munschauer M. Schwanhäusser B. Vasile A. Murakawa Y. Schueler M. (2012). The mRNA-Bound Proteome and its Global Occupancy Profile on Protein-Coding Transcripts. Mol. Cel. 46, 674–690. 10.1016/j.molcel.2012.05.021 22681889

[B6] Benjamini Y. Hochberg Y. (1995). Controlling the False Discovery Rate: A Practical and Powerful Approach to Multiple Testing. J. R. Stat. Soc. Ser. B (Methodological) 57, 289–300. 10.1111/j.2517-6161.1995.tb02031.x

[B7] Bolger A. M. Lohse M. Usadel B. (2014). Trimmomatic: a Flexible Trimmer for Illumina Sequence Data. Bioinformatics 30, 2114–2120. 10.1093/bioinformatics/btu170 24695404 PMC4103590

[B8] Buster D. W. Baird D. H. Yu W. Solowska J. M. Chauvière M. Mazurek A. (2003). Expression of the Mitotic Kinesin Kif15 in Postmitotic Neurons: Implications for Neuronal Migration and Development. J. Neurocytol. 32, 79–96. 10.1023/a:1027332432740 14618103

[B9] Carpenter S. Ricci E. P. Mercier B. C. Moore M. J. Fitzgerald K. A. (2014). Post-transcriptional Regulation of Gene Expression in Innate Immunity. Nat. Rev. Immunol. 14, 361–376. 10.1038/nri3682 24854588

[B10] Castello A. Fischer B. Eichelbaum K. Horos R. Beckmann B. M. Strein C. (2012). Insights into RNA Biology from an Atlas of Mammalian mRNA-Binding Proteins. Cell 149, 1393–1406. 10.1016/j.cell.2012.04.031 22658674

[B11] Castello A. Horos R. Strein C. Fischer B. Eichelbaum K. Steinmetz L. M. (2013). System-wide Identification of RNA-Binding Proteins by Interactome Capture. Nat. Protoc. 8, 491–500. 10.1038/nprot.2013.020 23411631

[B12] Chatterjee A. Dimitropoulou C. Drakopanayiotakis F. Antonova G. Snead C. Cannon J. (2007). Heat Shock Protein 90 Inhibitors Prolong Survival, Attenuate Inflammation, and Reduce Lung Injury in Murine Sepsis. Am. J. Respir. Crit. Care Med. 176, 667–675. 10.1164/rccm.200702-291OC 17615388 PMC1994236

[B13] Chatterjee A. Snead C. Yetik-Anacak G. Antonova G. Zeng J. Catravas J. D. (2008). Heat Shock Protein 90 Inhibitors Attenuate LPS-Induced Endothelial Hyperpermeability. Am. J. Physiology-Lung Cell Mol. Physiol. 294, L755–L763. 10.1152/ajplung.00350.2007 18245267

[B14] De Vries S. Naarmann-De Vries I. S. Urlaub H. Lue H. Bernhagen J. Ostareck D. H. (2013). Identification of DEAD-Box RNA Helicase 6 (DDX6) as a Cellular Modulator of Vascular Endothelial Growth Factor Expression under Hypoxia*. J. Biol. Chem. 288, 5815–5827. 10.1074/jbc.M112.420711 23293030 PMC3581395

[B15] Deng H. Maitra U. Morris M. Li L. (2013). Molecular Mechanism Responsible for the Priming of Macrophage Activation. J. Biol. Chem. 288, 3897–3906. 10.1074/jbc.M112.424390 23264622 PMC3567643

[B16] Dong Z. Wu S. Zhu C. Wang X. Li Y. Chen X. (2019). Clustered Regularly Interspaced Short Palindromic Repeats (CRISPR)/Cas9-mediated Kif15 Mutations Accelerate Axonal Outgrowth during Neuronal Development and Regeneration in Zebrafish. Traffic 20, 71–81. 10.1111/tra.12621 30411440 PMC6317882

[B17] Drechsler H. Mcainsh A. D. (2016). Kinesin-12 Motors Cooperate to Suppress Microtubule Catastrophes and Drive the Formation of Parallel Microtubule Bundles. Proc. Natl. Acad. Sci. USA 113, E1635–E1644. 10.1073/pnas.1516370113 26969727 PMC4812750

[B18] Echtenkamp F. J. Zelin E. Oxelmark E. Woo J. I. Andrews B. J. Garabedian M. (2011). Global Functional Map of the P23 Molecular Chaperone Reveals an Extensive Cellular Network. Mol. Cel. 43, 229–241. 10.1016/j.molcel.2011.05.029 PMC3155841 21777812

[B19] Felts S. J. Toft D. O. (2003). p23, a Simple Protein with Complex Activities. Cell Stress Chaper 8, 108–113. 10.1379/1466-1268(2003)008<0108:paspwc>2.0.co;22 PMC514861 14627195

[B20] Feng J. Hu Z. Chen H. Huan J. Wu R. Dong Z. (2016). Depletion of Kinesin-12, a Myosin-IIB Interacting Protein, Promotes Migration of Cortical Astrocytes. J. Cel. Sci. 129, 2438–2447. 10.1242/jcs.181867 PMC4920250 27170353

[B21] Ferenz N. P. Gable A. Wadsworth P. (2010). Mitotic Functions of Kinesin-5. Semin. Cel. Dev. Biol. 21, 255–259. 10.1016/j.semcdb.2010.01.019 PMC2844466 20109572

[B22] Gautier E. L. Shay T. Shay T. Miller J. Greter M. Jakubzick C. (2012). Gene-expression Profiles and Transcriptional Regulatory Pathways that Underlie the Identity and Diversity of Mouse Tissue Macrophages. Nat. Immunol. 13, 1118–1128. 10.1038/ni.2419 23023392 PMC3558276

[B23] Gomez-Rial J. Rivero-Calle I. Salas A. Martinon-Torres F. (2020). Role of Monocytes/Macrophages in Covid-19 Pathogenesis: Implications for Therapy. Infect. Drug. Resist. Vol. 13, 2485–2493. 10.2147/IDR.S258639 32801787 PMC7383015

[B24] Hara S. Kamei D. Sasaki Y. Tanemoto A. Nakatani Y. Murakami M. (2010). Prostaglandin E Synthases: Understanding Their Pathophysiological Roles through Mouse Genetic Models. Biochimie 92, 651–659. 10.1016/j.biochi.2010.02.007 20159030

[B25] Harrison R. E. Grinstein S. (2002). Phagocytosis and the Microtubule Cytoskeleton. Biochem. Cel. Biol. 80, 509–515. 10.1139/o02-142 12440692

[B26] Holt S. E. Aisner D. L. Baur J. Tesmer V. M. Dy M. Ouellette M. (1999). Functional Requirement of P23 and Hsp90 in Telomerase Complexes. Genes Dev. 13, 817–826. 10.1101/gad.13.7.817 10197982 PMC316592

[B27] Hotchkiss R. S. Karl I. E. (2003). The Pathophysiology and Treatment of Sepsis. N. Engl. J. Med. 348, 138–150. 10.1056/NEJMra021333 12519925

[B28] Hu J. Toft D. Anselmo D. Wang X. (2002). *In Vitro* reconstitution of Functional Hepadnavirus Reverse Transcriptase with Cellular Chaperone Proteins. J. Virol. 76, 269–279. 10.1128/jvi.76.1.269-279.2002 11739692 PMC135730

[B29] Jakobsson P.-J. Thoren S. Morgenstern R. Samuelsson B. (1999). Identification of Human Prostaglandin E Synthase: a Microsomal, Glutathione-dependent, Inducible Enzyme, Constituting a Potential Novel Drug Target. Proc. Natl. Acad. Sci. 96, 7220–7225. 10.1073/pnas.96.13.7220 10377395 PMC22058

[B30] Johnson J. L. Beito T. G. Krco C. J. Toft D. O. (1994). Characterization of a Novel 23-kilodalton Protein of Unactive Progesterone Receptor Complexes. Mol. Cel. Biol. 14, 1956–1963. 10.1128/mcb.14.3.1956 PMC358554 8114727

[B31] Kafasla P. Skliris A. Kontoyiannis D. L. (2014). Post-transcriptional Coordination of Immunological Responses by RNA-Binding Proteins. Nat. Immunol. 15, 492–502. 10.1038/ni.2884 24840980

[B32] Kahn O. I. Baas P. W. (2016). Microtubules and Growth Cones: Motors Drive the Turn. Trends Neurosciences 39, 433–440. 10.1016/j.tins.2016.04.009 PMC4930683 27233682

[B33] Kashina A. S. Baskin R. J. Cole D. G. Wedaman K. P. Saxton W. M. Scholey J. M. (1996). A Bipolar Kinesin. Nature 379, 270–272. 10.1038/379270a0 8538794 PMC3203953

[B34] Kharraz Y. Lefort A. Libert F. Mann C. J. Gueydan C. Kruys V. (2016). Genome-wide Analysis of TIAR RNA Ligands in Mouse Macrophages before and after LPS Stimulation. Genomics Data 7, 297–300. 10.1016/j.gdata.2016.02.007 26981431 PMC4778682

[B35] Kim D. Pertea G. Trapnell C. Pimentel H. Kelley R. Salzberg S. L. (2013). TopHat2: Accurate Alignment of Transcriptomes in the Presence of Insertions, Deletions and Gene Fusions. Genome Biol. 14, R36. 10.1186/gb-2013-14-4-r36 23618408 PMC4053844

[B36] Kishor A. White E. J. F. Matsangos A. E. Yan Z. Tandukar B. Wilson G. M. (2017). Hsp70's RNA-Binding and mRNA-Stabilizing Activities Are Independent of its Protein Chaperone Functions. J. Biol. Chem. 292, 14122–14133. 10.1074/jbc.M117.785394 28679534 PMC5572911

[B37] Kobayashi T. Nakatani Y. Tanioka T. Tsujimoto M. Nakajo S. Nakaya K. (2004). Regulation of Cytosolic Prostaglandin E Synthase by Phosphorylation. Biochem. J. 381, 59–69. 10.1042/BJ20040118 15040786 PMC1133762

[B38] Kratochvill F. Machacek C. Vogl C. Ebner F. Sedlyarov V. Gruber A. R. (2011). Tristetraprolin‐driven Regulatory Circuit Controls Quality and Timing of mRNA Decay in Inflammation. Mol. Syst. Biol. 7, 560. 10.1038/msb.2011.93 22186734 PMC3737733

[B39] Kwon S. C. Yi H. Eichelbaum K. Föhr S. Fischer B. You K. T. (2013). The RNA-Binding Protein Repertoire of Embryonic Stem Cells. Nat. Struct. Mol. Biol. 20, 1122–1130. 10.1038/nsmb.2638 23912277

[B40] Liao Y. Castello A. Fischer B. Leicht S. Föehr S. Frese C. K. (2016). The Cardiomyocyte RNA-Binding Proteome: Links to Intermediary Metabolism and Heart Disease. Cel. Rep. 16, 1456–1469. 10.1016/j.celrep.2016.06.084 PMC4977271 27452465

[B41] Liepelt A. Mossanen J. C. Denecke B. Heymann F. De Santis R. Tacke F. (2014). Translation Control of TAK1 mRNA by hnRNP K Modulates LPS-Induced Macrophage Activation. RNA 20, 899–911. 10.1261/rna.042788.113 24751651 PMC4024643

[B42] Liepelt A. Naarmann-De Vries I. S. Simons N. Eichelbaum K. Föhr S. Archer S. K. (2016). Identification of RNA-Binding Proteins in Macrophages by Interactome Capture. Mol. Cell Proteomics 15, 2699–2714. 10.1074/mcp.M115.056564 27281784 PMC4974345

[B43] Lin S. Liu M. Mozgova O. I. Yu W. Baas P. W. (2012). Mitotic Motors Coregulate Microtubule Patterns in Axons and Dendrites. J. Neurosci. 32, 14033–14049. 10.1523/JNEUROSCI.3070-12.2012 23035110 PMC3482493

[B44] Liu M. Nadar V. C. Kozielski F. Kozlowska M. Yu W. Baas P. W. (2010). Kinesin-12, a Mitotic Microtubule-Associated Motor Protein, Impacts Axonal Growth, Navigation, and Branching. J. Neurosci. 30, 14896–14906. 10.1523/JNEUROSCI.3739-10.2010 21048148 PMC3064264

[B45] Livak K. J. Schmittgen T. D. (2001). Analysis of Relative Gene Expression Data Using Real-Time Quantitative PCR and the 2−ΔΔCT Method. Methods 25, 402–408. 10.1006/meth.2001.1262 11846609

[B46] Mahboubi H. Moujaber O. Kodiha M. Stochaj U. (2020). The Co-chaperone HspBP1 Is a Novel Component of Stress Granules that Regulates Their Formation. Cells 9, 825. 10.3390/cells9040825 9 PMC7226807 32235396

[B47] Medzhitov R. Horng T. (2009). Transcriptional Control of the Inflammatory Response. Nat. Rev. Immunol. 9, 692–703. 10.1038/nri2634 19859064

[B48] Mi H. Muruganujan A. Casagrande J. T. Thomas P. D. (2013). Large-scale Gene Function Analysis with the PANTHER Classification System. Nat. Protoc. 8, 1551–1566. 10.1038/nprot.2013.092 23868073 PMC6519453

[B49] Miyakoshi L. M. Marques-Coelho D. De Souza L. E. R. Lima F. R. S. Martins V. R. Zanata S. M. (2017). Evidence of a Cell Surface Role for Hsp90 Complex Proteins Mediating Neuroblast Migration in the Subventricular Zone. Front. Cel. Neurosci. 11, 138. 10.3389/fncel.2017.00138 PMC5434112 28567003

[B50] Miyata Y. (2009). Protein Kinase CK2 in Health and Disease. Cell. Mol. Life Sci. 66, 1840–1849. 10.1007/s00018-009-9152-0 19387550 PMC11115779

[B51] Moritz B. Lilie H. Naarmann-De Vries I. S. Urlaub H. Wahle E. Ostareck-Lederer A. (2014). Biophysical and Biochemical Analysis of hnRNP K: Arginine Methylation, Reversible Aggregation and Combinatorial Binding to Nucleic Acids. Biol. Chem. 395, 837–853. 10.1515/hsz-2014-0146 25003387

[B52] Murakami M. Naraba H. Tanioka T. Semmyo N. Nakatani Y. Kojima F. (2000). Regulation of Prostaglandin E2 Biosynthesis by Inducible Membrane-Associated Prostaglandin E2 Synthase that Acts in Concert with Cyclooxygenase-2. J. Biol. Chem. 275, 32783–32792. 10.1074/jbc.M003505200 10869354

[B53] Naarmann I. S. Harnisch C. Muller-Newen G. Urlaub H. Ostareck-Lederer A. Ostareck D. H. (2010). DDX6 Recruits Translational Silenced Human Reticulocyte 15-lipoxygenase mRNA to RNP Granules. RNA 16, 2189–2204. 10.1261/rna.2211110 20884783 PMC2957058

[B54] Nair S. C. Toran E. J. Rimerman R. A. Hjermstad S. Smithgall T. E. Smith D. F. (1996). A Pathway of Multi-Chaperone Interactions Common to Diverse Regulatory Proteins: Estrogen Receptor, Fes Tyrosine Kinase, Heat Shock Transcription Factor Hsf1, and the Aryl Hydrocarbon Receptor. Cell Stress Chaper 1, 237–250. 10.1379/1466-1268(1996)001<0237:apomci>2.3.co;22 PMC376461 9222609

[B55] Naraba H. Murakami M. Matsumoto H. Shimbara S. Ueno A. Kudo I. (1998). Segregated Coupling of Phospholipases A2, Cyclooxygenases, and Terminal Prostanoid Synthases in Different Phases of Prostanoid Biosynthesis in Rat Peritoneal Macrophages. J. Immunol. 160, 2974–2982. 9510202

[B56] Ostareck D. H. Ostareck-Lederer A. (2019). RNA-binding Proteins in the Control of LPS-Induced Macrophage Response. Front. Genet. 10, 31. 10.3389/fgene.2019.00031 30778370 PMC6369361

[B57] Ostareck D. H. Ostareck-Lederer A. Wilm M. Thiele B. J. Mann M. Hentze M. W. (1997). mRNA Silencing in Erythroid Differentiation: hnRNP K and hnRNP E1 Regulate 15-Lipoxygenase Translation from the 3’ End. Cell 89, 597–606. 10.1016/s0092-8674(00)80241-x 9160751

[B58] Ostareck-Lederer A. Ostareck D. H. Cans C. Neubauer G. Bomsztyk K. Superti-Furga G. (2002). c-Src-mediated Phosphorylation of hnRNP K Drives Translational Activation of Specifically Silenced mRNAs. Mol. Cell. Biol. 22, 4535–4543. 10.1128/mcb.22.13.4535-4543.2002 12052863 PMC133888

[B59] Ostareck-Lederer A. Ostareck D. H. Standart N. Thiele B. J. (1994). Translation of 15-lipoxygenase mRNA Is Inhibited by a Protein that Binds to a Repeated Sequence in the 3’ Untranslated Region. EMBO J. 13, 1476–1481. 10.1002/j.1460-2075.1994.tb06402.x 8137829 PMC394967

[B60] Rao A. N. Baas P. W. (2018). Polarity Sorting of Microtubules in the Axon. Trends Neurosciences 41, 77–88. 10.1016/j.tins.2017.11.002 PMC5801152 29198454

[B61] Reynier F. De Vos A. F. Hoogerwerf J. J. Bresser P. Van Der Zee J. S. Paye M. (2012). Gene Expression Profiles in Alveolar Macrophages Induced by Lipopolysaccharide in Humans. Mol. Med. 18, 1303–1311. 10.2119/molmed.2012.00230 22952057 PMC3521791

[B62] Robinson M. D. Mccarthy D. J. Smyth G. K. (2010). edgeR: a Bioconductor Package for Differential Expression Analysis of Digital Gene Expression Data. Bioinformatics 26, 139–140. 10.1093/bioinformatics/btp616 19910308 PMC2796818

[B63] Rougerie P. Miskolci V. Cox D. (2013). Generation of Membrane Structures during Phagocytosis and Chemotaxis of Macrophages: Role and Regulation of the Actin Cytoskeleton. Immunol. Rev. 256, 222–239. 10.1111/imr.12118 24117824 PMC3806206

[B64] Rutledge H. R. Jiang W. Yang J. Warg L. A. Schwartz D. A. Pisetsky D. S. (2011). Gene Expression Profiles of RAW264.7 Macrophages Stimulated with Preparations of LPS Differing in Isolation and Purity. Innate Immun. 18, 80–88. 10.1177/1753425910393540 21239457

[B65] Scholey J. M. (2009). Kinesin-5 in Drosophila Embryo Mitosis: Sliding Filament or Spindle Matrix Mechanism? Cell Motil. Cytoskeleton 66, 500–508. 10.1002/cm.20349 19291760 PMC2778298

[B66] Sedlyarov V. Fallmann J. Ebner F. Huemer J. Sneezum L. Ivin M. (2016). Tristetraprolin Binding Site Atlas in the Macrophage Transcriptome Reveals a Switch for Inflammation Resolution. Mol. Syst. Biol. 12, 868. 10.15252/msb.20156628 27178967 PMC4988506

[B67] Semrad K. (2011). Proteins with RNA Chaperone Activity: a World of Diverse Proteins with a Common Task-Impediment of RNA Misfolding. Biochem. Res. Int. 2011, 1–11. 10.1155/2011/532908 PMC3017892 21234377

[B68] Sharova L. V. Sharov A. A. Nedorezov T. Piao Y. Shaik N. Ko M. S. H. (2009). Database for mRNA Half-Life of 19 977 Genes Obtained by DNA Microarray Analysis of Pluripotent and Differentiating Mouse Embryonic Stem Cells. DNA Res. 16, 45–58. 10.1093/dnares/dsn030 19001483 PMC2644350

[B69] Sleiman D. Bernacchi S. Xavier Guerrero S. Brachet F. Larue V. Paillart J.-C. (2014). Characterization of RNA Binding and Chaperoning Activities of HIV-1 Vif Protein. RNA Biol. 11, 906–920. 10.4161/rna.29546 25144404 PMC4179964

[B70] Smale S. T. (2012). Transcriptional Regulation in the Innate Immune System. Curr. Opin. Immunol. 24, 51–57. 10.1016/j.coi.2011.12.008 22230561 PMC3288296

[B71] Staudacher J. J. Naarmann-de Vries I. S. Ujvari S. J. Klinger B. Kasim M. Benko E. (2015). Hypoxia-induced Gene Expression Results from Selective mRNA Partitioning to the Endoplasmic Reticulum. Nucleic Acids Res. 43, 3219–3236. 10.1093/nar/gkv167 25753659 PMC4381074

[B72] Stoecklin G. Tenenbaum S. A. Mayo T. Chittur S. V. George A. D. Baroni T. E. (2008). Genome-wide Analysis Identifies Interleukin-10 mRNA as Target of Tristetraprolin. J. Biol. Chem. 283, 11689–11699. 10.1074/jbc.M709657200 18256032 PMC2431067

[B73] Stuart L. M. Ezekowitz R. A. B. (2005). Phagocytosis. Immunity 22, 539–550. 10.1016/j.immuni.2005.05.002 15894272

[B74] Tajima T. Murata T. Aritake K. Urade Y. Hirai H. Nakamura M. (2008). Lipopolysaccharide Induces Macrophage Migration via Prostaglandin D2and Prostaglandin E2. J. Pharmacol. Exp. Ther. 326, 493–501. 10.1124/jpet.108.137992 18492946

[B75] Tanikawa N. Ohmiya Y. Ohkubo H. Hashimoto K. Kangawa K. Kojima M. (2002). Identification and Characterization of a Novel Type of Membrane-Associated Prostaglandin E Synthase. Biochem. Biophysical Res. Commun. 291, 884–889. 10.1006/bbrc.2002.6531 11866447

[B76] Tanioka T. Nakatani Y. Kobayashi T. Tsujimoto M. Oh-Ishi S. Murakami M. (2003). Regulation of Cytosolic Prostaglandin E2 Synthase by 90-kDa Heat Shock Protein. Biochem. Biophysical Res. Commun. 303, 1018–1023. 10.1016/s0006-291x(03)00470-4 12684036

[B77] Tanioka T. Nakatani Y. Semmyo N. Murakami M. Kudo I. (2000). Molecular Identification of Cytosolic Prostaglandin E2 Synthase that Is Functionally Coupled with Cyclooxygenase-1 in Immediate Prostaglandin E2Biosynthesis. J. Biol. Chem. 275, 32775–32782. 10.1074/jbc.M003504200 10922363

[B78] Thangjam G. S. Dimitropoulou C. Joshi A. D. Barabutis N. Shaw M. C. Kovalenkov Y. (2014). Novel Mechanism of Attenuation of LPS-Induced NF-Κb Activation by the Heat Shock Protein 90 Inhibitor, 17-N-Allylamino-17-Demethoxygeldanamycin, in Human Lung Microvascular Endothelial Cells. Am. J. Respir. Cel. Mol. Biol. 50, 942–952. 10.1165/rcmb.2013-0214OC PMC4068943 24303801

[B79] Thomas P. D. Campbell M. J. Kejariwal A. Mi H. Karlak B. Daverman R. (2003). PANTHER: a Library of Protein Families and Subfamilies Indexed by Function. Genome Res. 13, 2129–2141. 10.1101/gr.772403 12952881 PMC403709

[B80] Tiedje C. Diaz-Muñoz M. D. Trulley P. Ahlfors H. Laass K. Blackshear P. J. (2016). The RNA-Binding Protein TTP Is a Global post-transcriptional Regulator of Feedback Control in Inflammation. Nucleic Acids Res. 44, gkw474–7440. 10.1093/nar/gkw474 PMC5009735 27220464

[B81] Vaure C. l. Liu Y. (2014). A Comparative Review of Toll-like Receptor 4 Expression and Functionality in Different Animal Species. Front. Immunol. 5, 316. 10.3389/fimmu.2014.00316 25071777 PMC4090903

[B82] Weaver A. J. Sullivan W. P. Felts S. J. Owen B. A. L. Toft D. O. (2000). Crystal Structure and Activity of Human P23, a Heat Shock Protein 90 Co-chaperone. J. Biol. Chem. 275, 23045–23052. 10.1074/jbc.M003410200 10811660

[B83] Weikl T. Abelmann K. Buchner J. (1999). An Unstructured C-Terminal Region of the Hsp90 Co-chaperone P23 Is Important for its Chaperone Function 1 1Edited by R. Huber. J. Mol. Biol. 293, 685–691. 10.1006/jmbi.1999.3172 10543959

[B84] Xu Z. Pal J. K. Thulasiraman V. Hahn H. P. Chen J.-J. Matts R. L. (1997). The Role of the 90-kDa Heat-Shock Protein and its Associated Cohorts in Stabilizing the Heme-Regulated Eif-24Al Kinase in Reticulocyte Lysates during Heat Stress. Eur. J. Biochem. 246, 461–470. 10.1111/j.1432-1033.1997.t01-1-00461.x 9208939

[B85] Zanotti S. Kumar A. Kumar A. (2002). Cytokine Modulation in Sepsis and Septic Shock. Expert Opin. Investig. Drugs 11, 1061–1075. 10.1517/13543784.11.8.1061 12150702

