# Supplementary material for: P23 Acts as Functional RBP in the Macrophage Inflammation Response
Source: Front Mol Biosci. 2021 Jun 11;8:625608. doi: 10.3389/fmolb.2021.625608 (PMC8226254; doi:10.3389/fmolb.2021.625608)

Supplementary Material

P23 acts as functional RBP in the macrophage inflammation response

Sebastian de Vries[1], Vladimir Benes[2], Isabel S. Naarmann-de Vries[1,#], Cornelia Rücklé[3,Δ], Katharina Zarnack[3], Gernot Marx[1], Dirk H. Ostareck[1] and Antje Ostareck-Lederer[1]

[1]Department of Intensive Care Medicine, University Hospital RWTH Aachen, Germany; [2]Genomics Core Facility, European Molecular Biology Laboratory (EMBL), Heidelberg, Germany, [3]Buchmann Institute of Molecular Life Science, Goethe University Frankfurt, Germany

present address:
[#]Section of Bioinformatics and Systems Cardiology, University Hospital Heidelberg, Germany
[Δ]Institute of Molecular Biology (IMB), Mainz, Germany

List of siRNAs, FISH probe, antibodies, primers

siRNAs

siRNA	Sequence	Reference
**si-P23-749**	AAGACAGTGATGATGAAAAGA	Rao et al., 2006
**si-P23-1672**	CTAATAAACTGTTGGAAGA	
**si-Kif15-544**	GCTCCTTTATTGAGGTCTA	
**si-Kif15-2481**	GCTGACAAAGAACTTTCTT	
**scr**	AGGUAGUGUAAUCGCCUUG	Naarmann et al., 2008

FISH probe

mRNA	Gene ID	Binding region
**Kif15**	209737	1452-1481

Antibodies for Western blot (WB) and immunofluorescence staining (IF)

Name	Company	Catalogue number	Dilution (WB)	Dilution (IF)
**VINCULIN**	Merck, Darmstadt, Germany	V9131	1:3000	
**GAPDH**	Abcam, Cambridge, UK	8245-100	1:5000	
**P23 (JJ3)**	Novus Biologicals, Centennial, CO, USA	NB 300-57	1:1000	1:200
**HSP90 (F-8)**	Santa Cruz, Dallas, TX, USA	sc-13119	1:200	
**KIF15**	Proteintech, Rosemont, IL, Texas	55407-1-AP	1:1000	
**CK2A1**	Proteintech, Rosemont, IL, Texas	10992-1-AP	1:1000	

qPCR primer

target	Primer	Sequence 5‘ – 3‘	Reference
**Luciferase firefly**	fw	CCTTCCGCATAGAACTGCCT	Liepelt et al., 2014
	rv	GGTTGGTACTAGCAACGCAC	
**Kif15**	fw	GACAAGGAACTTGTCACCAACCTC	
	rv	CATGTCCCTCTCGATGTCCTCTG	
**Kif1b**	fw	CTTTGAGCACACTGAGATACGC	
	rv	CAATAATATCTCCCAGTCCCTGAGC	
**Kif13a**	fw	CATAAAGCACAGTGATGACGAGG	
	rv	CAAACTCTGGGTGAAACTCTGC	
**Ndufv1**	fw	GCCTCCAATTTGCAGGTAGCTAT	Liepelt et al., 2014
	rv	CACGCACCACAAACACATCA	
**Tnf-α**	fw	TCCCCAAAGGGATGAGAAG	Liepelt et al., 2014
	rv	GCACCACTAGTTGGTTGTC	
**Il-1β**	fw	AAACCTTTGACCTGGGCTGTCCTG	Liepelt et al., 2014
	rv	AGAGGATGGGCTCTTCTTC	
**P23**	fw	CACCATGCAGCCTGCTTCTG	
	rv	TCGCTTCCTCCAAGACAACTG	
**Actb**	fw	CTCTAGACTTCGAGCAGGAGATGG	Borkham-Kamphorst et al., 2013
	rv	ATGCCACAGGATTCCATACCCAAGA	

References

Liepelt, A., Mossanen, J. C., Denecke, B., Heymann, F., De Santis, R., Tacke, F., Marx, G., Ostareck, D. H., and Ostareck-Lederer, A. (2014). Translation control of TAK1 mRNA by hnRNP K modulates LPS-induced macrophage activation. *RNA 20*, 899-911. DOI: 10.1261/rna.042788.113

Naarmann, I. S., Harnisch, C., Flach, N., Kremmer, E., Kuhn, H., Ostareck, D. H., and Ostareck-Lederer, A. (2008). mRNA silencing in human erythroid cell maturation: heterogeneous nuclear ribonucleoprotein K controls the expression of its regulator c-Src. *J. Biol. Chem. 283*, 18461-18472. DOI: 10.1074/jbc.M710328200

Rao, R. V., Niazi, K., Mollahan, P., Mao, X., Crippen, D., Poksay, K. S., Chen, S., and Bredesen, D. E. (2006). Coupling endoplasmic reticulum stress to the cell-death program: a novel HSP90-independent role for the small chaperone protein p23. *Cell Death Differ 13*, 415-425.
DOI: 10.1038/sj.cdd.4401761

Borkham-Kamphorst E., van de Leur E., Zimmermann H. W., Karlmark K. R., Tihaa L., Haas U., Tacke F., Berger T., Mak T. W., Weiskirchen R. (2013). Protective effects of lipocalin-2 (LCN2) in acute liver injury suggest a novel function in liver homeostasis. *Biochim Biophys Acta* 1832(5):660-73. DOI: 10.1016/j.bbadis.2013.01.014

Supplementary Figure 1. Multiple repetitions of the U/G-rich motif occur in the 3'UTRs of the P23-interacting mRNAs.

Colored boxes represent instances of the U/G-rich motif shown in Figure 3A, with height and color indicating *p*-value, as predicted by MAST. Only motifs with an *p*-value less than 0.0001 are shown (same settings as in MAST standard output). Ensembl gene ID and gene name are given for each gene. 3'UTRs are drawn to scale.

Supplementary Figure 1

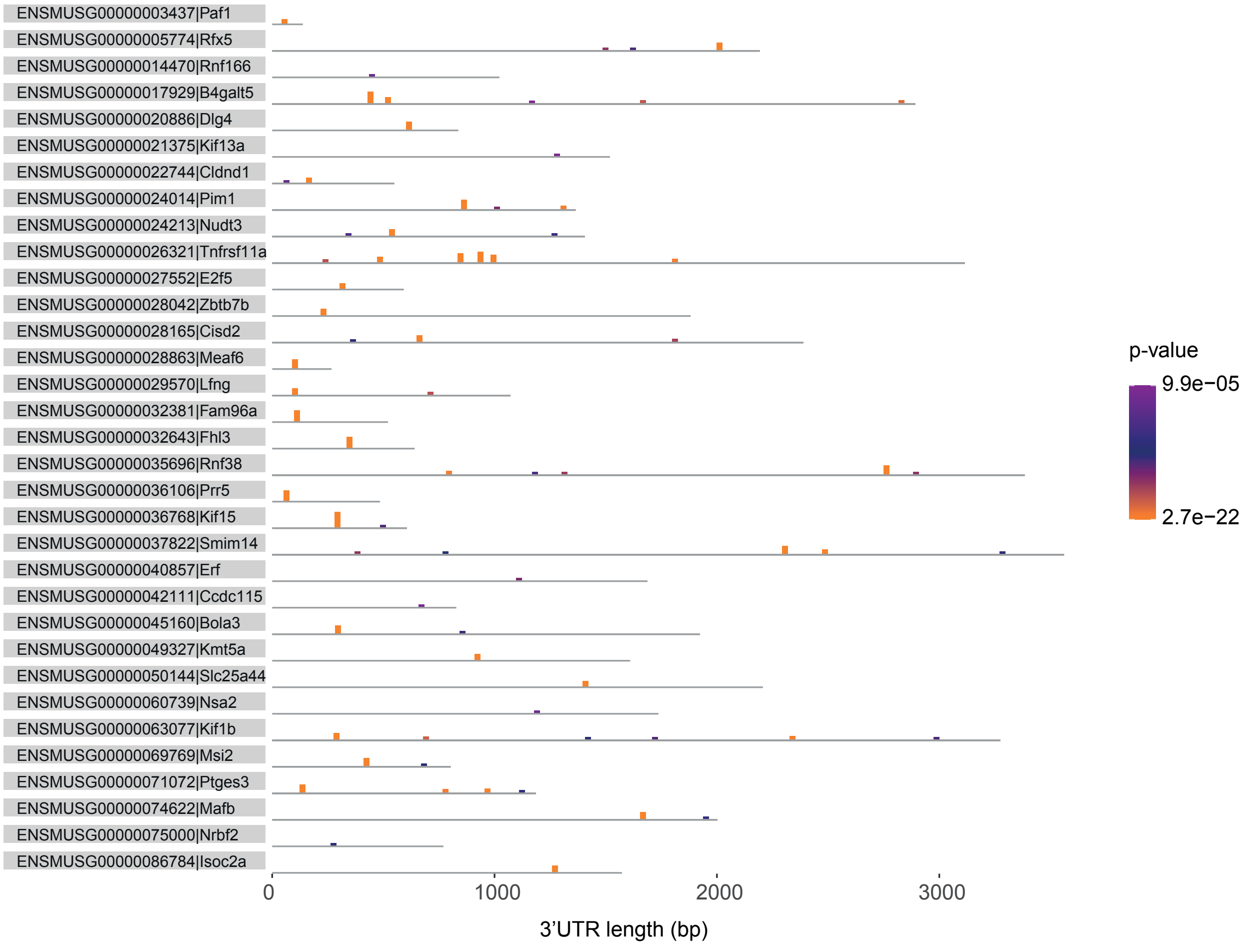

Supplement: Supplementary file 1 [file DataSheet1.PDF]
